# Genome-wide investigation of SnRK2 gene family in two jute species: *Corchorus olitorius* and *Corchorus capsularis*

**DOI:** 10.1186/s43141-022-00453-x

**Published:** 2023-01-18

**Authors:** Borhan Ahmed, Fakhrul Hasan, Anika Tabassum, Rasel Ahmed, Rajnee Hassan, Md. Ruhul Amin, Mobashwer Alam

**Affiliations:** 1 Basic and Applied Research On Jute Project, Bangladesh Jute Research Institute, Dhaka, 1207 Bangladesh; 2 Faculty of Agriculture, Bangabandhu Sheikh Mujibur Rahman Agricultural University, Salna, Gazipur, 1706 Bangladesh; 3 American International University of Bangladesh, Dhaka, 1229 Bangladesh; 4 Department of Biochemistry, University of Nebraska-Lincoln, Lincoln, NE USA; 5 Queensland Alliance for Agriculture and Food Innovation, The University of Queensland, 47 Mayers Rd, Nambour, QLD 4560 Australia

**Keywords:** SnRK2s, Phylogenetic analysis, Waterlogging, Drought, Salt tolerance, Expression analysis

## Abstract

**Background:**

Sucrose non-fermenting-1 (SNF1)-related protein kinase 2 (SnRK2), a plant-specific serine/threonine kinase family, is associated with metabolic responses, including abscisic acid signaling under biotic and abiotic stresses. So far, no information on a genome-wide investigation and stress-mediated expression profiling of jute SnRK2 is available. Recent whole-genome sequencing of two *Corchorus* species prompted to identify and characterize this SnRK2 gene family.

**Result:**

We identified seven SnRK2 genes of each of *Corchorus olitorius* (*Co*) and *C. capsularis* (*Cc*) genomes, with similar physico-molecular properties and sub-group patterns of other models and related crops. In both species, the SnRK2 gene family showed an evolutionarily distinct trend*.* Highly variable C-terminal and conserved N-terminal regions were observed. Co- and CcSnRK2.3, Co- and CcSnRk2.5, Co- and CcSnRk2.7, and Co- and CcSnRK2.8 were upregulated in response to drought and salinity stresses. In waterlogging conditions, Co- and CcSnRk2.6 and Co- and CcSnRK2.8 showed higher activity when exposed to hypoxic conditions. Expression analysis in different plant parts showed that SnRK2.5 in both *Corchorus* species is highly expressed in fiber cells providing evidence of the role of fiber formation.

**Conclusion:**

This is the first comprehensive study of SnRK2 genes in both *Corchorus* species. All seven genes identified in this study showed an almost similar pattern of gene structures and molecular properties. Gene expression patterns of these genes varied depending on the plant parts and in response to abiotic stresses.

**Supplementary Information:**

The online version contains supplementary material available at 10.1186/s43141-022-00453-x.

## Background

Sucrose non-fermenting 1 (SNF1)-related kinase (SnRKs) is a type of protein kinase, which is involved in stress tolerance signaling pathways in plants. SnRKs are categorized as SnRK1, SnRK2, and SnRK3 based on their domain structure, sequence homology, and their roles in cellular functions [1]. SnRK2s, which are members of adenosine monophosphate (AMP)-activated protein kinase (AMPK), are only found only in plants. They play critical roles in response to abiotic stresses and are involved in abscisic acid (ABA)-mediated signaling pathways [2–4]. However, some SnRK2s regulate seed germination, seedling growth, maturation, flowering time, seed dormancy, and stomatal movement during drought conditions [5–8]. Therefore, these proteins are critical growth and development of plants.

SnRK2s have SNF1/AMP kinases like conserved N-terminal catalytic domains that are essential for kinase activity. The C-terminal domain of this protein contains stretches of acidic amino acids, either glutamic acid (group I), or aspartic acid (groups II and III) [9]. Two subdomains, domain I and domain II, constitute the C-terminal domain. Domain I is present in all SnRK2 family members and is located 20 amino acids away from the catalytic domain. Domain II is required for ABA response and is exclusive to group III [10–12]. In general, ABA substantially activates SnRK2 group III members, slightly induces group II members, and moderately stimulates group I [10–14]. SnRK2’s ability to physically engage with type 2C protein phosphatase (PP2Cs), an important component of the ABA signaling pathway, relies on its adaptable and changeable C-terminal domain [3, 5, 9, 15].

The function of SnRK2s in stress signaling pathways has been studied on a range of horticultural and industrial crops, including rice [14], maize [12], sorghum [16], apple [17], pakchoi [18], grape [19], rubber [20], sugarcane [21] pepper [22], sugar beet [23], and cotton [24]. Investigations on *Arabidopsis thaliana* identified that all the *AtSnRK2s* except *AtSnRK2.9* are stimulated by different osmolytes, indicating their general response to osmotic stress [1, 10, 25]. Among these, *AtSnRK2.2/2.3/2.6* play a critical role in the ABA signal transduction network in response to environmental stresses [3, 11]. All of the SnRK2s in *Oryza sativa* (named *OsSAPK1-OsSAPK10*) are activated by hyperosmotic stress, and three of them (*OsSAPK8/9/10*) are activated by ABA [13]. *OsSAPK4* has been reported to control genes involved in oxidative stress response functioning and ion homoeostasis [26]. Increased expression of *AtSnRK2.8* and *OsSAPK4* in transgenic plants resulted in a significant improvement in tolerance to salt and drought stress [26, 27]. Overexpression of *AtSnRK2.6* significantly boosted the levels of carbon and energy demanding physiological activities in Arabidopsis leaves, such as fatty acid and sucrose metabolism [28]. Furthermore, genes from the wheat SnRK2 subfamily, such as *TaSnRK2.4*, *TaSnRK2.7*, and *TaSnRK2.8*, were used to promote tolerance to multi-abiotic stressors in Arabidopsis by upregulating their expression [29–31]. Overexpression of sugarcane *SoSnRK2.1* was found to improve drought tolerance in tobacco [32]. All of these evidences of the SnRK2 gene family’s participation in response to multiple environmental stresses clearly demonstrated that this family can possibly be employed for crop genetic improvement, particularly for abiotic stress tolerance [33, 34].

Jute (*Corchorus* sp.) is a major source of natural fiber, accounting for 80% of global bast fiber production [35]. Despite the fact that the Malvaceae family contains over 100 species, there are only two commercially grown Corchorus species, *C. olitorius* and *C. capsularis*. Jute is basically self-pollinated and has 14 diploid chromosomes (2*n* = 14). The genome sizes of *C. olitorius* and *C. capsulris* are 445.05 Mb and 338.13 Mb, respectively [36]. Since 2017, several transcriptomic data have been generated by multiple projects in Bangladesh and China [36–39]. These data sets created an opportunity for comparative genomic studies to identify and characterize key gene families for future genetic improvement of this crop.

Jute is particularly susceptible to abiotic stress. Due to climate change, temperature and rainfall have been fluctuating in recent years, and as a result, jute production has decreased [40]. In addition, salt stress created a deleterious impact on jute development and physiological characteristics, resulting in worse yield quantity and quality [41]. Drought diminishes fiber yield by 20 to 30% and lowers fiber quality [42, 43]. Waterlogging stress is a problem for jute, especially at the seedling stage [44, 45]. Other stresses, including toxic metals, extreme temperatures, and too much light, are also detrimental to jute development and production [46, 47]. As the SnRK2 family of genes plays an important role in abiotic stress resistance [2, 10, 17], jute is a cash crop, in order to secure their food farmers’ fertile arable land for food crop production, and jute is pushed into marginal or stressed-prone areas. The SnRK2 gene plays an important role against abiotic stress, such as salt, drought, and waterlogging. In jute, CDPK [48] and FLA [49] gene families were well studied, but there is a lack of information regarding SnRK2. Identification and characterization of the SnRK2 gene family in both jute species (*C. olitorius* and *C. capsularis*) might help in downstream research for the desired improvement of this biodegradable fiber. In this study, we conducted a comparative genomic study of both jute species to explore gene structures and functions, phylogenetic relationships, and expression patterns against salt stress. Findings from this study provide an essential understanding of SnRK2 genes in jute and constitute a foundation for further investigation to use them in genetic improvement program.

## Methods

### Genomic data mining and SnRK2 gene identification

To identify the SnRK2 protein sequences in *C. capsularis* and *C. olitorius*, reference proteins of well-established SnRK2 protein sequences were chosen as query sequences. The validated reference proteins of *Arabidopsis* are downloaded from TAIR database (http://www.arabidopsis.org); *Theobroma cacao* and *Gossypium raimondii* from phytozome (http://phytozome.jgi.doe.gov/) and both *Corchorus* sp. Genome sequence from the Basic and Applied Research on Jute Project (www.jutegenome.org) [36]. The downloaded sequences were used as a query to perform BLAST [48] with the jute genome database and identified the putative homolog in the jute species. A cutoff *e* value of e^−10^ was used for the identification of the candidate SnRK2 genes. The amino acid sequence of candidate genes was analyzed to examine the presence of the characteristic serine/threonine protein kinases domain (PF00069) using Pfam (http://pfam.xfam.org/) [49]. All output genes were manually checked, and the predicted genes lacking serine/threonine protein kinases domains were rejected.

### Nomenclature and classification

The nomenclature of the identified jute SnRK2 was applied following the nomenclature guidelines for SnRK2 [21]; genes were grouped based on the phylogenetic tree and homology to AtSnRK2 [50, 51] (Table 1). Here, *Co* and *Cc* prefixes were used for *C. olitorius* and *C. capsularis*, respectively. Orthologs of phylogenetically related SnRK2 genes of the same clade with the model plant *Arabidopsis*, and each pair of paralogous genes with a high percentage of similarity in the amino acid sequence was used for classification and nomenclature [10].

**Table 1:** Chromosomal locations of validated CoSnRK2 and CcSnRK2 genes

Sl. No	Gene name	Protein ID	Chromosome/scaffold	Scaffold	Chromosome/ scaffolD position	Contig	Contig position
**a. *Corchorus olitorius***							
1	CoSnRK2.3	COLO4_11351	LG5	scf7180001685701	160,113–162,639	contig14721	58,976–61,502
2	CoSnRK2.4a	COLO4_04355	scf7180001681417	scf7180001681417	17,566–21,079	contig11423	17,566–21,079
3	CoSnRK2.4b	COLO4_11768	LG5	scf7180001685702	262,907–265,304	contig14773	223,636–226,033
4	CcSnRK2.5	COLO4_06686	scf7180001685069	scf7180001685069	42,836–45,718	contig12876	26,118–29,000
5	CoSnRK2.6	COLO4_11725	LG5	scf7180001685702	66,575–69,499	contig14773	27,304–30,228
6	CoSnRK2.7	COLO4_27287	LG1	scf7180001685868	1,963,664–1,966,276	contig19559	81,066–83,678
7	CoSnRK2.8	COLO4_12024	scf7180001685704	scf7180001685704	555,262–558,012	contig14830	12,736–15,486
**b. *Corchorus capsularis***							
1	CcSnRK2.3	CCACVL1_26314	LG5	scf7180000535437	10,170,301–10,172,677	contig14484	7701–10,077
2	CcSnRK2.4a	CCACVL1_16888	LG2	scf7180000535334	6,446,492–6,449,084	contig11152	62,104–64,696
3	CcSnRK2.4b	CCACVL1_06930	scf7180000534926	scf7180000534926	38,039–41,920	contig08274	31,143–35,024
4	CcSnRK2.5	CCACVL1_00490	scf7180000534656	scf7180000534656	201,997–205,942	contig01234	1559–5504
5	CcSnRK2.6	CCACVL1_16921	LG2	scf7180000535334	6,621,296–6,624,224	contig11154	93,932–96,860
6	CcSnRK2.8	CCACVL1_26729	LG5	scf7180000535437	13,375,253–13,382,337	contig14552	35,479–42,563
7	CcSnRK2.7	CCACVL1_05979	scf7180000534755	scf7180000534755	89,489–90,928	contig07866	315–1754

To classify the jute SnRK2 gene family, phylogenetic analysis of both *Corchorus* genome and *Arabidopsis thaliana* was conducted using MUSCLE (https://www.ebi.ac.uk/Tools/msa/muscle/) [52] with the default settings. The bootstrap consensus phylogenetic tree, inferred from 1000 replicates, was constructed using the maximum likelihood (ML) [53] method in MEGA7 [54]. The naming of CoSnRK2 and CcSnRK2s genes were assigned according to the phylogenetic tree showing orthology with *Arabidopsis* along with their reciprocal BLASTP identity.

### Chromosomal location and gene structure analysis

The physical location of CoSnRK2 and CcSnRK2 genes on each *Corchorus* species chromosome/scaffold was detected using BLASTNT search against the local database of both jute genomes. The locations of the genes on the chromosome or the scaffold were indicated based on the starting position of all CoSnRK2 and CcSnRK2 genes. Scaffolds of the assembled sequences were used to locate tandem duplications as the chromosome-scale assembly is unavailable for both jute species.

Clustal Omega was utilized for multiple sequence alignment (https://www.ebi.ac.uk/Tools/msa/clustalo/) [55], which was visualized with ESPript (http://espript.ibcp.fr/ESPript/ESPript/) [56]. The protein module, based on *AtSnRK2.3* (PDB ID: 3UJG), was depicted and edited using SWISS-MODEL (http://swissmodel.expasy.org/) and Swiss-Pdb Viewer (http://spdbv.vital-it.ch/TheMolecularLevel/SPVTut/), respectively.

In silico approaches were performed to obtain the genomic sequences of the identified SnRK2 genes. A web application named Gene Structure Display Server using GSDS (http://gsds.cbi.pku.edu.cn/) [57] was accessed to determine the structure diagrams of the exon and intron of SnRK2 genes. Genome-wide molecular evolution study of the SnRK2 gene family was done by Multiple Expectation Maximization for motif Elicitation (MEME) utility program using the MEME online server (http://meme.sdsc.edu/meme/meme.html) [58].

### Characterization of SnRK2 gene family in jute

Assessment of the physical and chemical properties of the SnRK2 gene family proteins was performed by ProtParam online (http://expasy.org/tools/protparam.html) [59] to characterize for related indexes, including theoretical isoelectric point (pI), molecular weight, formula, aliphatic index, instability index, and grand average of hydropathicity (GRAVY). In order to predict the subcellular localization of CoSnRK and CcSnRK2s, Plant-mPloc server (http://www.csbio.sjtu.edu.cn/bioinf/plant-multi/) [60] was used. Secondary structures were predicted with the online SOPMA server (https://npsa-prabi.ibcp.fr/cgi-bin/npsa_automat.pl?page=/NPSA/npsa_sopma.html). Tertiary protein structures were predicted with the Phyre2 server (http://www.sbg.bio.ic.ac.uk/~phyre2/html/page.cgi?id = index). Disordered regions were predicted with the PONDR server (http://www.pondr.com/). The BetaCavityWeb server (http://voronoi.hanyang.ac.kr/betacavityweb) was used to search and analyze the channel structure. Web Procheck (https://saves.mbi.ucla.edu/) was used to predict Ramachandan plot structures.

### GO analysis of identified SnRK2 genes

Amino acid sequences are used as the input of Blast2GO program [61] with default parameters to conduct Gene Ontology (GO) annotation of CoSnRK2s and CcSnRK2s for describing the biological processes, cellular components, and molecular functions. Blast2GO utilized the output files of InterProScan and BLASTP to annotate GO categories and generate respective figures.

### Promoter analysis of identified SnRK2 genes

To identify putative *cis*-acting regulatory elements in the promoter sequences of the identified SnRK2 family genes, 1Kbp upstream intergenic region from the initiation codon (ATG) of the predicted transcription sites was extracted from jute genome data (www.jutegenome.org). The PlantCARE (http://bioinformatics.psb.ugent.be/webtools/plantcare/html/) [62] and PLACE (http://www.dna.affrc.go.jp/PLACE/) [63] databases were used to confirm the putative *cis*-elements in the promoters.

### Expression analysis

Sequence Read Archive (SRA) (https://www.ncbi.nlm.nih.gov/sra) was used to download publicly available transcriptome data, which were further analyzed to check the expression pattern of CcSnRK2 and CoSnRK2 genes in different tissue as well as under waterlogging, drought and salinity stress conditions. The accession numbers and sample data that were used in this study are listed in Table S1. The available transcriptome data of Yueyuan No.5 (YY) (*C. capsularis*) [39] and TC (*C. olitorius*) [38] which are two salt-tolerant varieties and accession NY/253C (NY) (*C. olitorius*) [38] which is sensitive to salinity were explored to reveal the gene expression pattern of leaf and root sample at 9 leaf stage with and 250-mM NaCl for salinity stress. On the other hand, the transcriptome data of drought-sensitive *C. capsularis* (Yueyuan No.5, YY) [37] and drought-tolerant *C. olitorius* (Gangfengchangguo, GF) [37] were treated with Polyethylene Glycol (PEG) at 9 leaf stage for performing drought stress study. Waterlogging stress data were generated through concurrent bioprojects, PRJNA215141 and PRJNA215142 (Table S1), which were previously generated by our group. Genes associated with fiber development and their expression pattern were explored using the transcriptomic data of seedlings and fiber cells of two jute species (*C. olitorius* var. O4; *C. capsularis* var. CVL-1) (Accession id: SRX2369402, SRX2369404, SRX2369401, SRX2369403) [36].

FastQC v.0.11.9 (Andrews S 2010) was used to check the quality parameters of all the transcriptome sequencing data generated with the Illumina sequencing platform. Low-quality reads found in the raw data were trimmed using Trimmomatic *v.*0.36 [64]. Mapping of the high-quality clean RNA-Seq reads to both *Corchoru*s species was done by TopHat2 (version 2.1.0, Baltimore, MD, USA) with the default parameters [65]. Then, Cufflinks2 v.2.1.1 suite [66] utilized the mapped reads to generate transcriptome assembly and perform differential expression analysis. The *p *values for differentially expressed genes (DGE) were calculated byCuffdiff2 based on the normalized fragments per kilobase of exon per million fragments mapped (FPKM) values. The heatmap function of R package (version 3.2.2; available online: https://cran.r-project.org/web/packages/pheatmap/) was exploited to generate clustered heatmap of *Z*-scaled FPKM values of CoSnRK2s and CcSnRK2s*.*

## Results

### Identification of SnRK2 genes with their physico-molecular properties

A total of seven candidate SnRK2 family proteins having complete serine/threonine protein kinase catalytic domains have been detected in both *Corchorus* species genome (Table 1). The information on gene names, gene ID, protein length, molecular weights (MW), isoelectric points (pI) GRAVY, instability index, and subcellular locations were listed in Table 2. The predicted length of 7 CoSnRK2 and 7 CcSnRK2 proteins ranged from 313 to 364 aa and 314 to 364 aa, correspondingly to the molecular weight ranges from 35.76 to 41.26 kDa and 35.87 to 41.26 KDa (Table 2). The predicted isoelectric point (pI) of all SnRK2 proteins (less than 7) attested that SnRK2 proteins were rich in acidic amino acids resembling all other plant species. The GRAVY score of all SnRK2 proteins was found to be negative possessing their hydrophilic nature (Table 2). Prediction of a subcellular location indicated that all SnRK2 proteins localized in the nucleus (Table 2).

**Table 2:** Details of SnRK2 genes identified from the genome-wide search analysis. The table shows the following details: putative SnRK2 gene name, protein length (number of amino acids), isoelectric point (pI), molecular formula, Aliphatic index, Instability index, GRAVY, predicted molecular mass, and subcellular localization in *Corchorus olitorius* (a) and *Corchorus capsularis* (b)

Sl no	Gene name	Protein length	Theorical pI	Molecular formula	Aliphatic index	Instability index	GRAVY	Molecular weight (Kda)	Subcellular localization
**a. *Corchorus olitorius***									
1	CoSnRK2.3	352	4.70	C_1765_H_2754_N_476_O_541_S_21_	86.99	38.32	− 0.275	39,971.41	Nucleus
2	CoSnRK2.4a	329	5.58	C_1665_H_2607_N_465_O_508_S_13_	78.81	50.5	− 0.56	37,683.61	Nucleus
3	CoSnRK2.4b	334	5.76	C_1693_H_2672_N_468_O_512_S_13_	82.87	53.79	− 0.511	38,191.45	Nucleus
4	CoSnRK2.5	343	5.56	C_1713_H_2713_N_469_O_520_S_13_	84.43	38.81	− 0.387	38,615.00	Nucleus
5	CoSnRK2.6	364	4.84	C_1821_H_2846_N_494_O_566_S_17_	88.9	42.57	− 0.34	41,260.62	Nucleus
6	CoSnRK2.7	313	4.96	C_1596_H_2472_N_426_O_484_S_12_	84.38	45.43	− 0.39	35,756.47	Nucleus
7	CoSnRK2.8	340	5.44	C_1727_H_2688_N_460_O_514_S_16_	85.97	34.54	− 0.288	38,632.07	Nucleus
**b. *Corchorus capsularis***									
1	CcSnRK2.3	361	4.73	C_1806_H_2820_N_486_O_554_S_21_	87.78	39.45	− 0.264	40,878.44	Nucleus
2	CcSnRK2.4a	320	5.76	C_1628_H_2546_N_456_O_493_S_13_	77.69	50.7	− 0.583	36,811.66	Nucleus
3	CcSnRK2.4b	342	5.35	C_1734_H_2707_N_475_O_527_S_14_	82.37	52.27	− 0.473	39,089.28	Nucleus
4	CcSnRK2.5	343	5.56	C_1714_H_2715_N_469_O_521_S_13_	84.14	38.81	− 0.394	38,645.02	Nucleus
5	CcSnRK2.6	364	4.84	C_1821_H_2846_N_494_O_566_S_17_	88.9	42.57	− 0.34	41,260.62	Nucleus
6	CcSnRK2.8	337	5.52	C_1712_H_2664_N_456_O_507_S_16_	86.74	35.2	− 0.258	38,259.70	Nucleus
7	CcSnRK2.7	314	4.96	C_1600_H_2478_N_428_O_486_S_12_	84.11	45.32	− 0.4	35,870.57	Nucleus

### Sequence alignment, phylogenetic analysis, and naming of SnRK2s

**Fig. 1:**
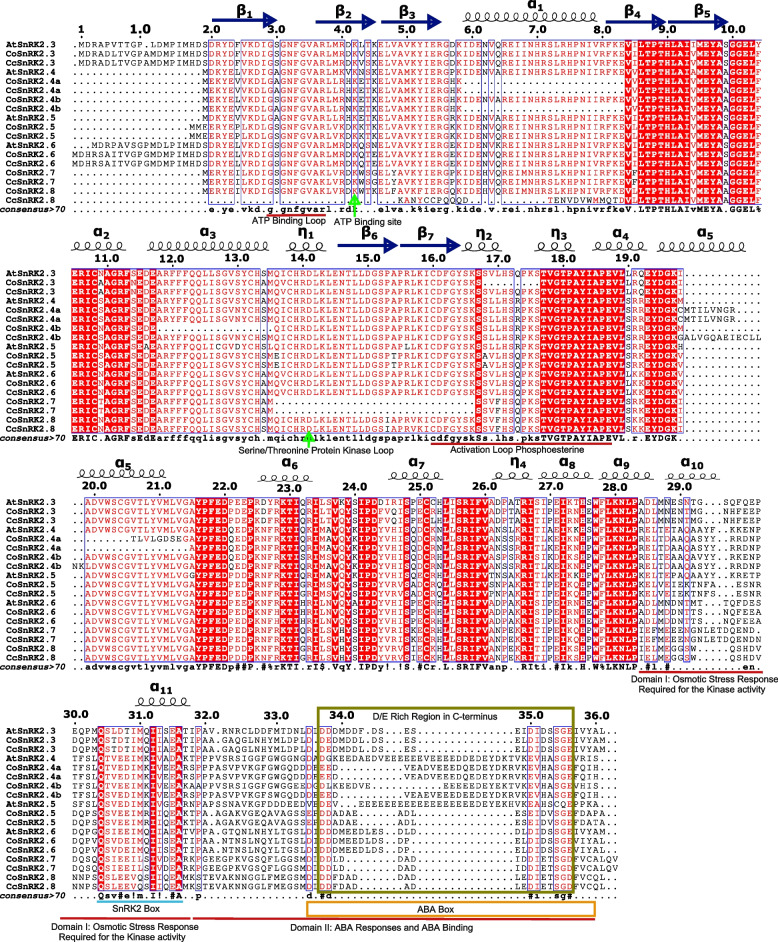
Structure-based alignment of the amino acid sequence of CoSnRK2 and CcSnRK2 with AtSnRK2.3, AtSnRK2.4, AtSnRK2.5, and AtSnRK2.6. Red background shows sequence identity and red letters show sequence similarity in the alignment. Secondary structure elements and ATP-binding loop, ATP-binding site, serine/threonine protein kinase loop, activation loop of phosphoesterine, SnRK2 box, and ABA box are indicated

The homology search by multiple sequence alignment of CoSnRK2 and CcSnRK2 proteins depicted the information of conserved motifs and domains having the conserved pattern of amino acid residues in Fig. 1. Sequence alignment indicated that CoSnRK2 and CcSnRK2 have the potential for serine/threonine and tyrosine kinase activities. It was observed that the CoSnRK2 and CcSnRK2s have highly conserved N-terminal catalytic domains and divergent C-terminal regulatory domains containing acidic amino acid-rich regions. All the SnRK2 proteins have two conserved signatures in their N-terminal regions- an ATP-binding loop having the amino acid pattern I/LGXGXFGVA and an ATP-binding site with a lysine residue (purple underline) (Fig. 1). The serine/threonine protein kinase active-site signature V/ICHRDLKLENTLL with an aspartic acid residue (the active site ↑) in all SnRK2s except *CoSnRK2.7* and *CcSnRK2.7*. The aspartic acid, serine (represent for proton acceptor active site), and phosphorine (brown underline) were highly conserved in all SnRK2s. The C-terminal domain consisted of two subdomains. Domain I which contained SnRK2-specific box from Gln-303 to Pro-318 was required for activation by osmotic stress and belonged to all the members of SnRK2s while on the contrary, and domain II was present only in both species *SnRK2.7* and *2.8* (olive box). Secondary structure prediction revealed that CoSnRK2 and CcSnRK2 formed eleven α-helixes and eight β-plated sheets.

**Fig. 2:**
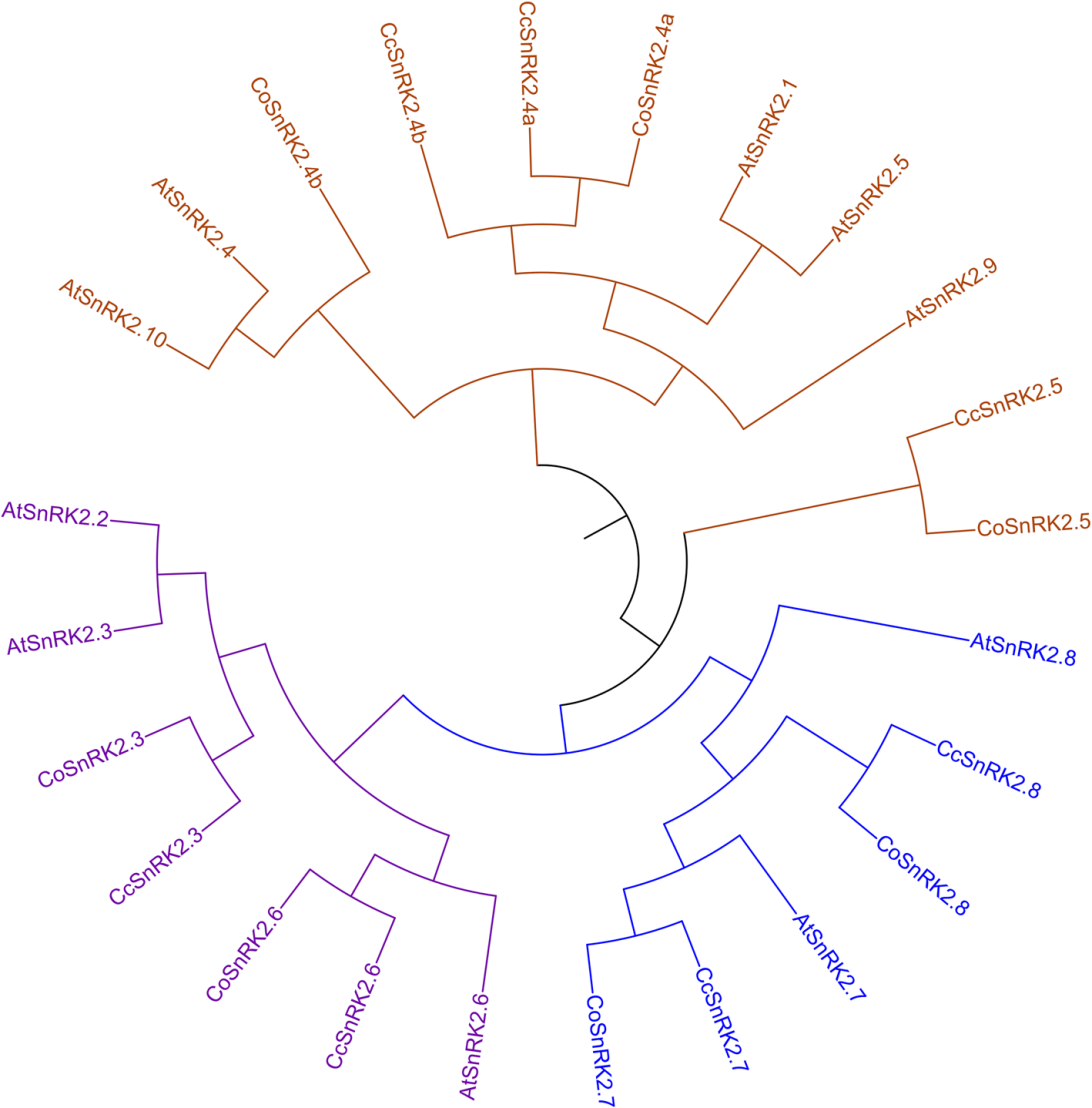
Phylogenetic relationship among the *C. olitorius*, *C. capsularis*, and *Arabidopsis thaliana* SnRK2 genes. The phylogeny was generated by MEGA7 with maximum likelihood (ML) method and 1000 replicates bootstraps, based on the amino acid sequence of 7, 7, and 10 SnRK2 genes from *C. olitorius*, *C. capsularis*, and* A. thaliana*, respectively. The gene name with purple, blue, and dark red represents SnRK2 groups 1, II, and III, respectively

To investigate the evolutionary relationship of SnRK2 among *Arabidopsis thaliana*, *Corchorus capsularis* and *Corchorus olitorius*, a phylogenetic tree was constructed using protein sequence of AtSnRK2s, CcSnRK2s, and CoSnRK2s (Additional file 1 and Fig. 2). Like SnKR2 in *Arabidopsis*, CoSnRK2 and CcSnRK2 proteins were clustered into three distinct subgroups, namely Groups 1–3 as shown in Fig. 2. In each group, the CoSnRK2 and CcSnRK2 have two or more orthologous members in AtSnRK2s. Group 1 including *AtSnRK2.2*,* AtSnRK2.3*, and *AtSnRK2.6* have been reported to be activated by ABA and involved in ABA signal transduction [67]. *AtSnRK2.8* falling in group 2 was found to improve the drought tolerance [27] of transgenic *Arabidopsis* and to participate in the metabolic process [68]. In *Corchorus olitorius CoSnRK2.3*,* CoSnRK2.6* and *Corchorus capsularis CcSnRK2.3*,* CcSnRK2.6* belonged to group 1. Group 2 comprised *SnRK2.7* and *2.8* in both jute species*.* There were 3 members of each jute species namely *SnRK2.4a*, *SnRK2.4b*, and *SnRK2.5* in group 3 (Fig. 2). Similar topological phylogenetic tree was observed in *Arabidopsis*, *Vitis vinifera*, *Brassica rapa*, Oryza sativa, *Saccharum officinarum*, and *Zea mays*, but there is another smallest clade (group4) found in *Malus domestica *[17] and *Glycine max* [17, 69].

In accordance to the nomenclature guidelines for SnRK2 [21], the nomenclature of CoSnRK2 and CcSnRK2 was applied according to the phylogenetic tree and homology to AtSnRK2 because it is easy for the readers to distinguish the *Arabidopsis* homolog. A two-letter prefix derived from the genus and species names of the organisms in which the genes are present, for example At for *Arabidopsis thaliana* [50, 51] was applied for the nomenclature of SnRK2 genes in both *Corchorus* species (Table 1). *Co* and *Cc* prefixes were used for *Corchorus olitorius* and *Corchorus capsularis*, respectively, where orthologs of phylogenetically related SnRK2 genes of the same clade with the model plant *Arabidopsis*, and each pair of paralogous genes has a high percentage of similarity in amino acid sequence (Table S2) was used for classification and nomenclature [10].

### Chromosomal distribution, gene structure, and conserved motif analysis

Seven SnRK2 family genes of each *Corchorus* species were mapped to the assembled genome of respective species, and genes were found to be distributed on different chromosomes along with scaffolds (Table 1). In *C. olitorius*, Chr5 contains three genes, Chr1 contains 1 gene, and rest three genes were found in different scaffolds. On the other hand, *C. capsularis* Chr2 and Chr5 contained two genes each and the rest three genes were observed in three different scaffolds.

**Fig. 3:**
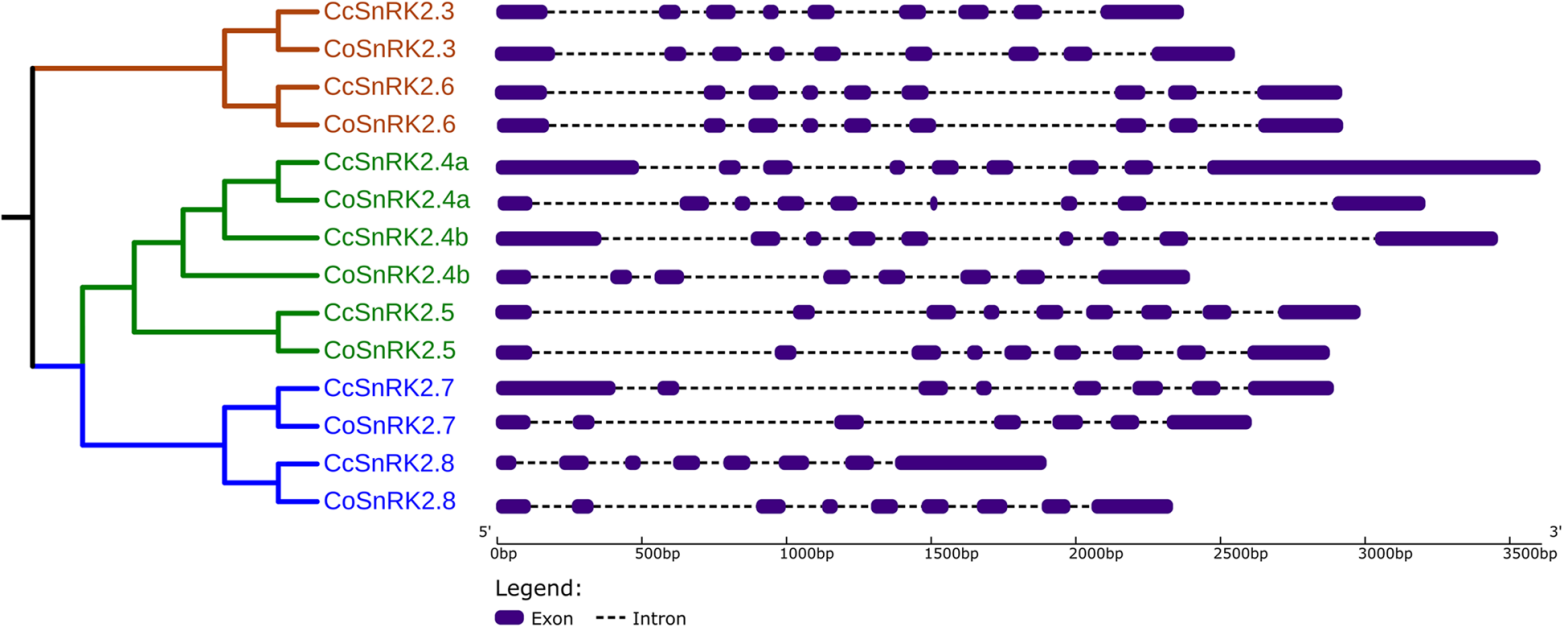
Exon–intron structure of 14 SnRK2s genes of *C. olitorius* and *C. capsularis* according to the phylogenetic tree. A graphic representation of the gene model was identified in this study. Exons are shown as dark blue boxes and introns are shown as brown dot lines

Analysis of the exon–intron structure of SnRK2 genes in two *Corchorus* species provides the evolutionary trajectory of this gene family. We have determined the distribution of the predicted exon–intron structure using coding regions of all SnRK2 genes from two *Corchorus* species in accordance with the phylogenetic tree (Fig. 3). In contrast to phylogenetic analysis, most members within the same group showed similar intron–exon structure and gene length. This conservation of exon and intron number in each group strongly supports the close evolutionary relationship of CcSnRK2 and CoSnRK2 genes. In addition, SnRK2 genes in both species are different groups and usually vary in intron phase pattern and gene length (Fig. 3).

Huai et al. [12] have reported that most of the SnRK2 from higher plants show a conserved distribution of exon and intron and have nine exons. Here, all the recognizable *Corchorus* species SnRK2 had eight introns that were different notably in size but had relatively conserved orders and approximate size of exon among them. The gene with nine exons has strictly conserved exon lengths. The length of the second through the eight exons were 75, 102, 54, 93, 93, 105, and 99, respectively (Fig. 3, Table S3). All identified *Corchorus* spp SnRK2 genes have nine exons except *CoSnRK2.4b* and *2.7* and *CcSnRK2.7* and *2.8*. Furthermore, the exon length and intron position of SnRK2 genes between two *Corchorus* species are remarkably similar. We found that the intron length of SnRK2 in both *Corchorus* species is generally much longer than exon.

The SOPMA web server was used to analyze secondary structures. The findings demonstrate that beta turns, extended strands, random coils, and -helices were all found in the ranges of 34.68 to 45.31%, 12.79 to 16.77%, 4.08 to 6.65%, and 34–43 to 47.77%, respectively (Table S4). The high quality and reliability of the protein structures were shown by a Ramachandran plot where the percentage of residues in the core, allowed, and generous regions all exceeded 79% (Table S4 and Fig. S1). In both the Co and CcSnRK2 proteins, the predicted channel structures varied from 1 to 8, with an overall percentage of disordered regions ranging from 17.61 to 39.36% (Table S4).

**Fig. 4:**
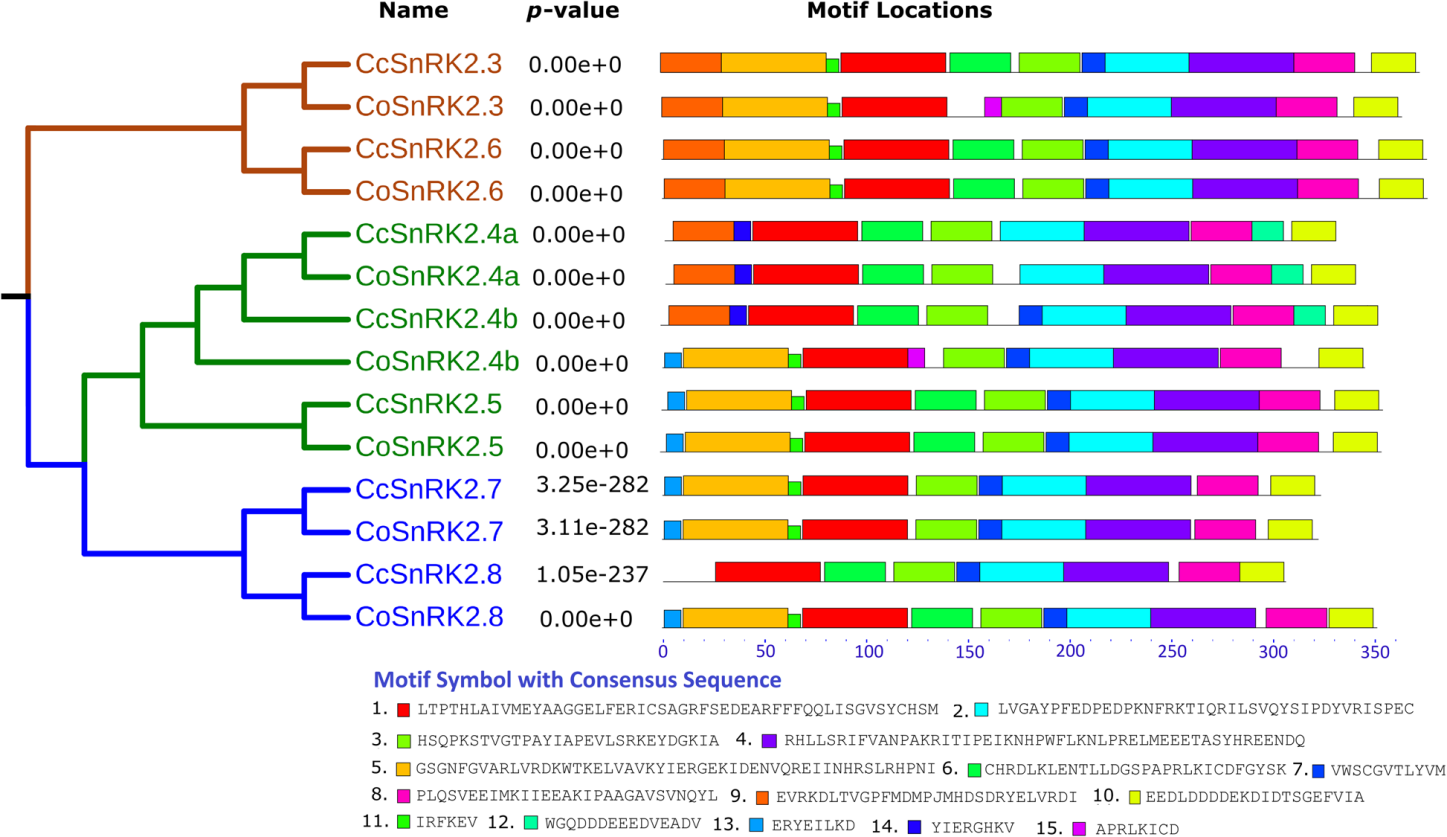
Motif analysis of jute SnRK2s according to the phylogenetic tree. All motifs were identified by the MEME database with complete amino acid sequences of *CoSnRK2s* and *CcSnRK2s*. The length of the motif for each SnRK2s protein is displayed proportionately

The CoSnRK2 and CcSnRK2s were highly conserved N-terminal kinase domains but divergent C-terminal domains. We employed MEME to detect conserved motifs in the CoSnRK2 and CcSnRK2 family and found fifteen conserved motifs and with their multilevel consensus sequence (Fig. 4). In general, most of the closely related members within the same clade had similar motif composition. All of the CoSnRK2 and CcSnRK2 proteins shared the same six designated motifs—1–3, 5, 7, 10. Motifs 6 and 7 also occurred in all SnRK2 sequences except for *CoSnRK2.7*,* CcSnRK2.7* and *CoSnRK2.4a*, respectively. In addition, motif 9 in the N-terminal peptide was conserved in both Corchorus species *SnRK2.3*,* SnRK2.6 SnRK2.4a*, and *CoSnRK2.4b* while C-terminal domain region which was rich in aspartate (D) and glutamine (E) acidic patch, [70] was conserved in all SnRK2s (Fig. 1).

### Gene ontology annotation and putative cis-element analysis of SnRK2 genes in jute

**Fig. 5:**
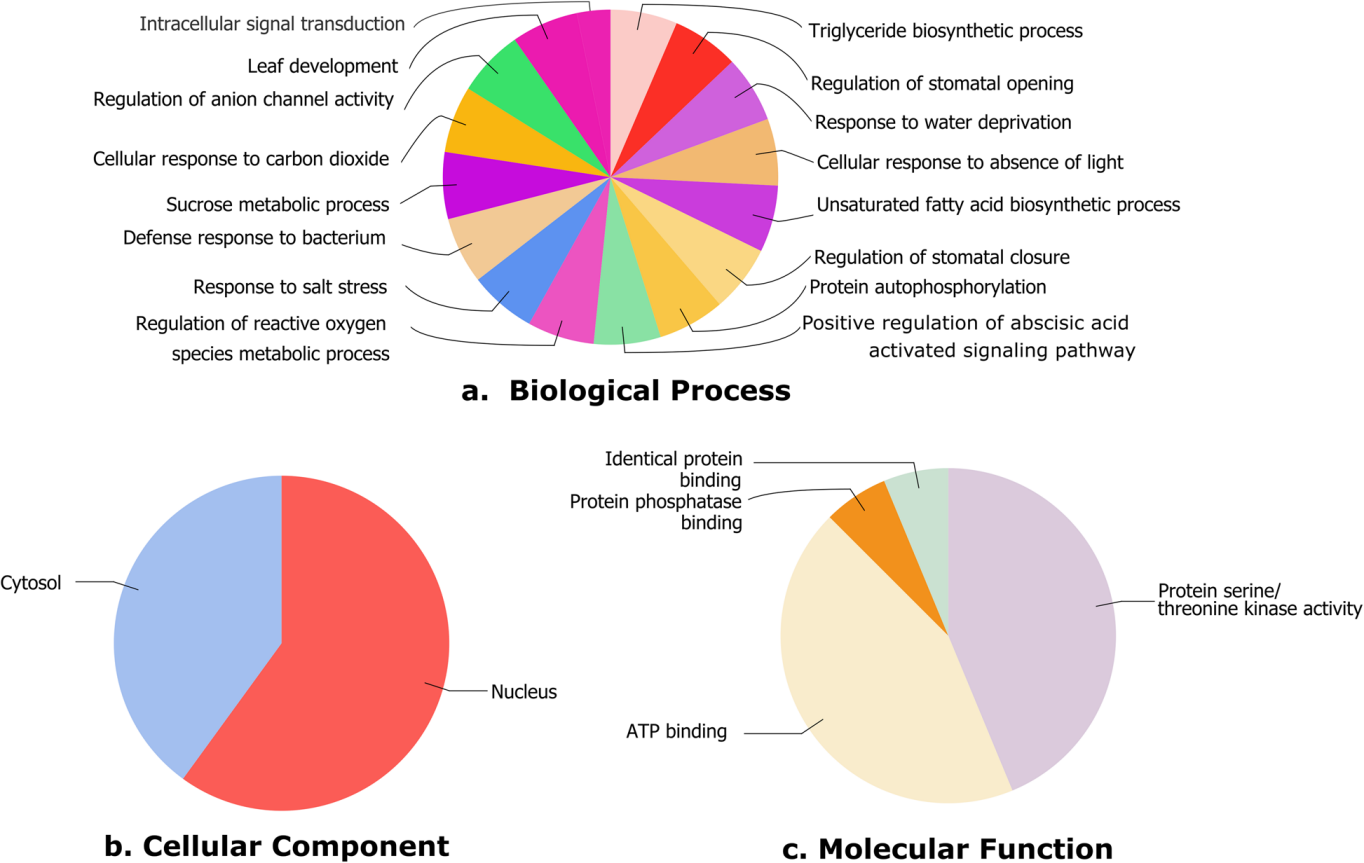
Gene ontology of CoSnRK2 and CcSnRK2 proteins biological process (**a**). Cellular components (**b**). Molecular function (**c**)

The Gene Ontology (GO) analysis was performed for specifying cellular location, molecular function, and diverse biological process participation of all the CoSnRK and CcSnRK2 proteins according to the GO database (Fig. 5 and Table S5). The analysis of biological processes revealed that the proteins were significantly associated with phosphorylation (GO:0,006,468), response to salt stress (GO:0,009,651), sucrose metabolic process (GO:0,005,985), response to water deprivation (GO:0,009,414), and other 12 different molecular functions (Fig. 5a and Table S5). The result also indicated that CoSnRK and CcSnRK2s are mainly localized in the nucleus (GO:0,005,634), along with in cytosol (GO:0,005,829) (Fig. 5b and Table S5). The molecular function clearly showed ATP binding (GO:0,005,524) and protein serine/threonine kinase activity (GO:0,004,674) were the main activities along with slightly protein phosphatase binding (GO:0,019,903), identical protein binding (GO:0,042,802) (Fig. 5c and Table S5). In conclusion, functional analysis of CoSnRK and CcSnRK2s suggested their involvement in diverse mechanisms with ATP binding and kinase activity initiated from the nuclear region.

### Promoter analysis of SnRK2 genes in both jute species

**Fig. 6:**
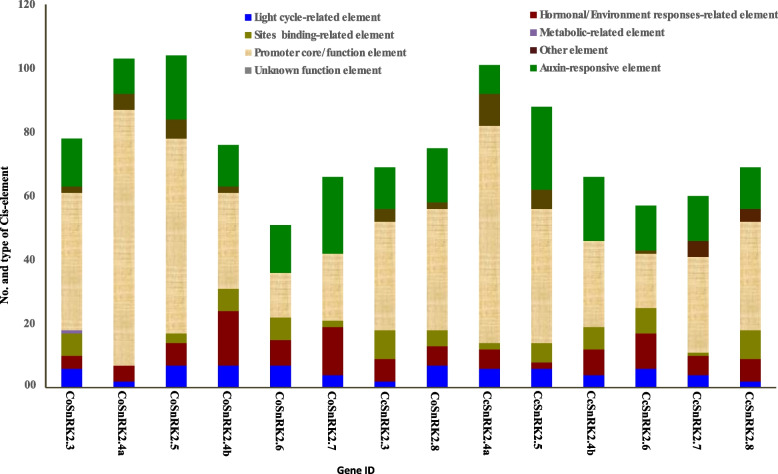
Promoter analysis of CoSnRK2 and CcSnRK2 genes. The numbers of different cis-elements were presented in the form of bar graphs; Different cis-elements with the same or similar functions are shown in the same color

*Cis*-regulatory elements not only control gene expression but also provide an initial trigger for the functional dissection of transcriptional sites in the upstream regions. To investigate the possible roles of SnRK2s identified in both jute genomes, corresponding promoter regions (1 kb in length upstream region from the initiation codon ATG) of the CoSnRK2 and CcSnRK2s genes were subjected to *cis*-element analysis by PlantCare and PLACE database (Fig. 6, Tables S6 and S7). Using the PlantCare database, we identified a total of 67 cis-element (three unnamed) in the promoter regions of SnRK2 genes (Table S6). The identified cis-elements were divided into eight major groups, such as hormonal/environment responsive (ARE, AuxRR core, ABRE, CGTCA motif, TCA, WUN motif, etc.), light responsive (GT1 motif, GATA motif, G-box, GATA motif, Box 4, etc.), site-binding-related element (Myb, MBS, CCAAT-box, MRE, AT-rich element, etc.), and promoter core functional element (TATA-box, TATA, CAAT-box) (Fig. 6, Table S6). The core promoter TATA-box and CAAT box had a greater number of promoter functions followed by unknown functions and site binding-related elements. The well-known stress–response element (STRE, AAGGGG) and ABA responsible element (ABRE, C/TACGTGGC) were observed in almost all the CoSnRK2s and CcSnRK2s showing their response against multiple stress, including cold, drought, and salt [71, 72]. CGTCA-motif and the TGACG-motif were involved in Methylejasmonate (MeJA) production in response to several environmental stresses. MeJA was found to be expressed in multiple physiological processes, including plant growth and development, abscission, maturity, and secondary metabolism [73–77].

On the other hand, a total of 188 cis-element found in the CoSnRK2s and CcSnRK2s using the PLACE database. Among them, around 40 elements were almost found in all the SnRK2s (Table S7). The most abundant core promoter elements (CRE) in all SnRK2 genes promoter was DOFCOREZM, which are specific DNA-binding proteins associated with the expression of multiple genes in plants. Besides this, a number of *cis*-elements were rich in both jute species, such as ABRELATERD1, ACGTATERD1, CCAATBOX1, GT1GMSCAM4, MYB1AT, and MYB2CONSENSUSAT elements related to abiotic stress, ABRERATCAL acted as Ca^2+^ responsive, -10PEHVPSBD, IBOX, IBOXCORE, IBOXCORENT, SORLIP1AT, BOXIIPCCHS involved in light and circadian rhythms regulation, ARR1AT, ABREOSRAB21, ACGTABREMOTIFA2OSEM, ASF1MOTIFCAMV related to phytohormone and GT1GMSCAM4, WBOXNTERF3 identified as pathogen-related.

### Expression profiles of the SnRK2 genes under abiotic stress

**Fig. 7:**
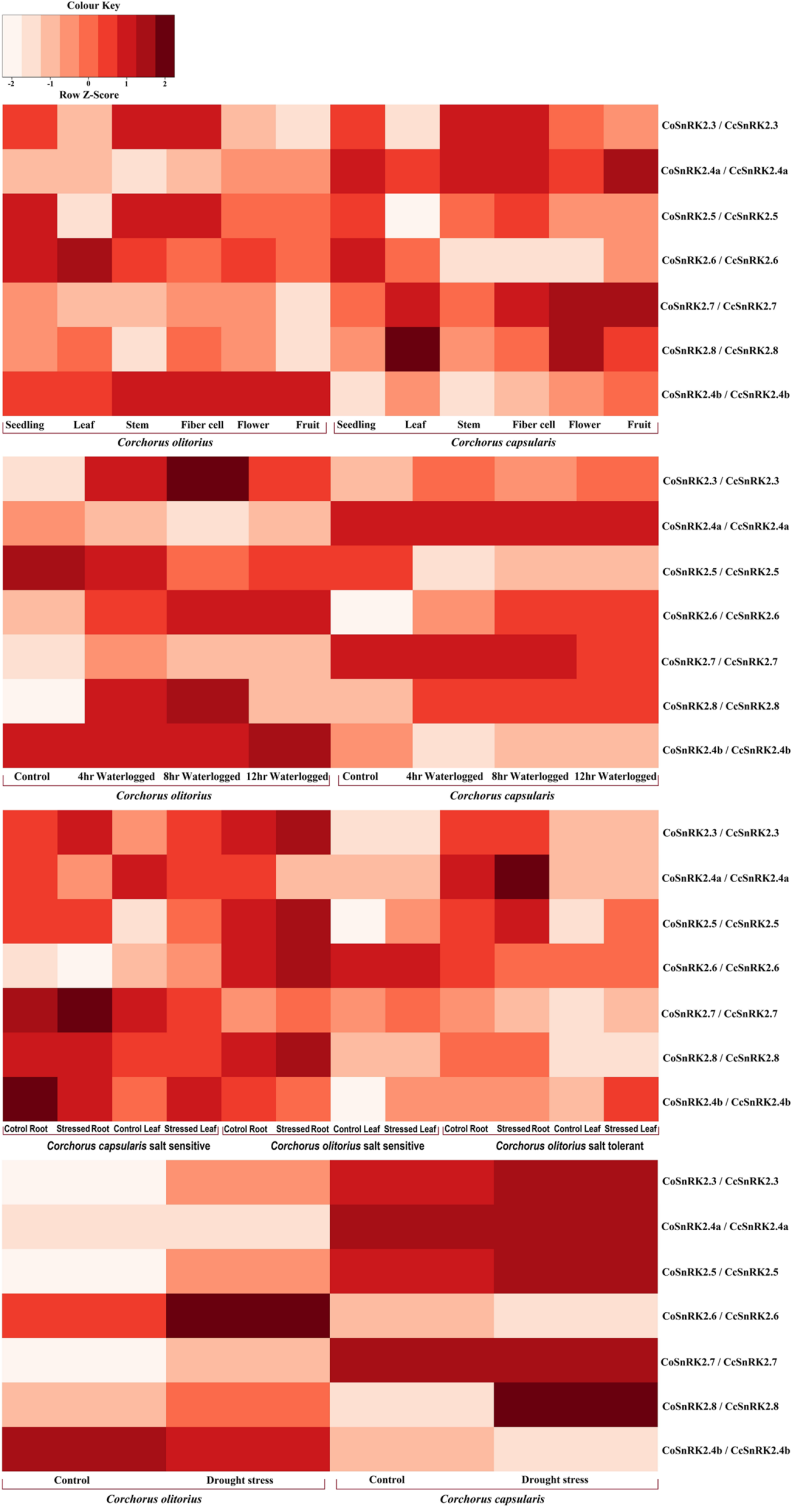
Expression profiling of CoSnRK2 and CcSnRK2 genes in six different tissues and against waterlogged stress, salt stress, and drought stress. The log2-transformed FPKM (fragments per kilobase of exon model per million mapped reads) added by a pseudo-count of 1 value was used for heat map construction

Gene expression profile provides an important clue to delineate gene functionality. The members of the SnRK2 gene family play important role in response to various environmental stresses such as higher osmotic stress, high salinity, and drought condition. In case of jute seedling and fiber, transcriptome data revealed that all the SnRK2 genes are expressed in both transcriptomes but *SnRK2.7 SnRK2.8* and *SnRK2.3* in both species showed higher expression in fiber than seedling (Fig. 7a; Table S8a). In case of waterlogging stress, most of the SnRK2 genes in both species showed higher expression over the stress period and then finally decline their expression to avoid energy losses for survival smoothly (Fig. 7b, Table S8b). But *SnRK2.3*,* 2.8* showed higher expression over time of waterlogging. The expression pattern of CcSnRK2.4b showed higher expression, whereas in CoSnRK2.4b, the expression is decreased over time. On the other hand, abiotic stress (salinity and drought) transcriptome data showed that *SnRK2.3*,* SnRK2.7*, and *SnRK2.8* in both jute species are upregulated under drought conditions in both species (Fig. 7c, d and Table S8c-d). In case of salinity-stressed transcriptome data, the *SnRK2.3* and *2.5* showed higher expression in both jute species in both root and leaf data. On the other hand, *SnRK2.7* and *SnRK2.8* in both species showed upregulation in the root system and downregulation in the leaf. In addition, salinity-stressed transcriptome showed a high expression of the *SnRK2.4b* in the leaf but a lower expression in the root.

## Discussion

The SnRK2 is composed of plant-specific small proteins, which play active roles in response to environmental stress specially salinity and drought [5–7, 9, 78]. This protein kinase family has been identified in many plant species, whereas little is known in *Corchorus* species. In this study, we identified, characterized, and observed the expression of SnRK2 genes in different plant parts, and under waterlogging, salinity, and drought stresses through a comprehensive genome-wide investigation on two *Corchorus* species. Results from this study provided valuable information for future genetic improvement of this fiber crop for adverse environmental conditions, developing new cultivars with improved fiber quality, and productivity will contribute to further industry expansion.

### Identification and characterization of SnRK2 genes in both Corchorus species

Our genome-wide analysis revealed 7 putative complete SnRK2 sequences in both *Corchorus* species. However, the number of identified SnRK2 genes varies depending on plant species. The number of SnRK2 genes in jute is almost one third of that of another fiber crop, *Gossypium hirsutum*, and slightly lower than in *Vitis venifera* (8 SnRK2) as well as in some diploid plant species (10 SnRK2), namely—*Arabidopsis thaliana*, *Saccharum officinarum*,* Oryza sativa*,* Hevea brasiliensis*, and* Sorghum bicolor*, higher than in *Carica papaya* (6 SnRK2), *Beta vulgaris* (6 SnRK2), and *Nicotiana tabacum* (3 SnRK2) (Table S9)*.* This variation in the number of SnRk2 genes could be due to the whole genome duplication events after the separation of plant lineage [79]. The predicted isoelectric point (pI) indicated that CoSnRK2 and CcSnRK2 proteins were rich in acidic amino acids and hydrophilic in nature and localized in the nucleus.

### Evolutionary relationships

Phylogenetic analysis and sequence alignment provide information on the evolutionary relationship of proteins of a gene family. To investigate the evolutionary relationship of SnRK2 among *Arabidopsis thaliana*, *C. capsularis*, and *C. olitorius*, a phylogenetic tree was constructed using a protein sequence of AtSnRK2s, CcSnRK2s, and CoSnRK2s. Like SnKR2 in *Arabidopsis*, CoSnRK2 and CcSnRK2 proteins were clustered into three distinct subgroups (Fig. 2), each containing two or more orthologous members. CoSnRK2 and CcSnRK2 genes of group 1 (*CoSnRK2.3*,* CoSnRK2.6*, *CcSnRK2.3*, and *CcSnRK2.6*) clustered with *AtSnRK2.2*,* AtSnRK2.3*, and *AtSnRK2.6*, which have been reported to be activated by ABA and involved in ABA signal transduction [67]. Jute SnRK2 in group 2 (*SnRK2.7* and *2.8* in both species) showed a strong evolutionary relationship with *AtSnRK2.8*, which was found to improve drought tolerance [27] and participate in metabolic processes [68]. Three members of each of CoSnRK2 and CcSnRK2 genes (*SnRK2.4a*, *2.4b*, and *2.5*) formed group 3 (Fig. 2). Similar topological phylogenetic tree was observed in *Arabidopsis* [10, 67], *Vitis vinifera* [19], *Brassica rapa* [18], *Oryza sativa* [67], *Saccharum officinarum* [21], and *Zea mays* [12], but another smallest clade (group4) was found in *Malus domestica* [69] and *Glycine max* [69]. Similarities of these orthologous genes clearly suggested that monocot and dicot divergence happened after the generation of SnRK2 genes.

The homology search by multiple sequence alignment of CoSnRK2 and CcSnRK2 proteins depicted the information of conserved motifs and domains having the conserved pattern of amino acid residues. In plants, SnRK2 contains two typical domains: a highly conserved N-terminal protein kinase and a variable C-terminal domain. Extensive evidence indicated that the C-terminal domain plays role in the functional diversity of SnRK2s [3, 9, 80]. Conserved motif analysis showed an uneven distribution of ten motifs in *CcSnRK2s* and *CoSnRK2s* sequences. Among these, motifs 1, 2, 4, and 6 can be found in all members, motifs 5 and 9 were only present in subclasses I, motif 7 was specific to groups II and III, and motifs 3 and 8 were unique to subclass III, suggesting that they might contribute to the functional specificity of corresponding groups. However, further studies are required on the motif-exchange experiment using protein interaction assays. SnRK2s are monomeric plant-specific Ser/Tr protein kinases with a molecular weight of approximately 40 kDa [81]. Group III members namely *AtSnRK2.2/2.3/2.6* have been systematically studied in *Arabidopsis*, and their structural profiles have been well characterized [6, 82]. Based on the amino acid sequence alignment and structural profile of *AtSnRK2.3/2.6* and CoSnRK and CcSnRK2s, some of the key segments near the N-terminal have been identified to contribute to basal activities (including ATP-binding loop, ATP-binding site, proton acceptor activate site, activation loop, and phosphoserine site). Furthermore, the α-helix and β-bridge were highly conserved. Sequence segments of the SnRK2 box, ABA box, and functional domains (domain I and domain II) that can be found near the C-terminal, were highly diversified in each sequence, which is in accordance with previous research of the functional diversity of SnRK2s known to be closely related to their C-terminal motifs [3, 9, 80].

The exon–intron organizations of CcSnRK2 and CoSnRK2 genes exhibited high similarity with *Arabidopsis thaliana* and rice [67]. Though the exon phasing of both jute species SnRK2s was highly conserved, the sizes of their introns varied a lot. All other CoSnRK2 and CcSnRK2 genes contained nine exons, except for *CoSnRK2.7* containing seven exons and *CcSnRK2.7*,* CoSnRK2.4b* and *CcSNRK2.8* containing eight exons. The similar distribution of eight introns and nine exons was also found in other species, such as *Arabidopsis thaliana*, rice, cotton, and maize, indicating the evolutionary conservation of gene structure of SnRK2s in higher plants [10, 12, 14, 17, 67].

### Jute SnRK2 genes are involved in abiotic stress resistance

The role of SnRK2 genes in several stress response has been demonstrated in numerous studies. The biological functions of CoSnRK2 and CcSnRK2s under abiotic stresses are still unknown. The comparative genomic analysis between *Arabidopsis* and jute provides information for studying and understanding the biological functions of CcSnRK2 and CoSnRK2s. It has been suggested that *AtSnRK2.7* and *AtSnRK2.8*, which are orthologous to *SnRK2.7* and *SnRK2.8* in both jute species, regulate some drought-responsive genes involving AREB/ABF in *Arabidopsis *[83]. Furthermore, it has been considered that *AtSnRK2.3* and *2.6*, orthologous to both Corchorus species *SnRK2.3* and *2.6* in both jute species respectively, are master regulators of the ABA signaling network to protect plants against abiotic stresses such as drought and salinity [82].

Gene expression patterns can provide important clues to gene functions, which are believed to be associated with divergence in the promoter region [84]. Cis-acting regulatory elements contained in gene’s promoter regions play key roles in conferring the developmental and environmental regulation of gene expression. In silico sequence analysis showed that the promoter of each gene contained an important putative cis-acting element, such as the ABA-response elements (ABREs) denoting possible ABA-dependent regulation [85], dehydration-responsive elements (MBS DRE/CRT and G/ACCGCC), low-temperature responsive elements (LTRE and CCGAC), heat shock elements (HSEs), and cis-elements necessary for induction of many heat shock-induced genes [86].

Re-analysis of public RNA-Seq datasets, we found that SnRK2 genes exhibited diverse expression patterns against drought and saline conditions [37–39]. The transcriptome data showed different and temporally dynamic expression patterns of CoSnRK2, and CcSnRK2 was under salt and drought stress. In case of jute seedling and fiber, transcriptome data revealed that all the SnRK2 genes were expressed in both transcriptome, but *SnRK2.7*,* SnRK2.8*, and *SnRK2.3* in both species showed higher expression in fiber than seedling indicating a role of fiber development. In waterlogged stress, the expression pattern of most genes decline over the period of time except SnRK2.3 and 2.8 in both species. In Arabidopsis, AtSnRK2.3 also showed its involvement in water stress response mainly in leaves [11]. It is evident that the *C. capsularis* is tolerated against waterlogging conditions, whereas *C. oiltorius* is susceptible in water stress [87] and CcSnRK2.4b expression is higher over the period of time but CoSnRK2.4b expression reduce over the period of time. Again, abiotic stress (salinity and drought) transcriptome data revealed that *SnRK2.3*,* SnRK2.7*, and *SnRK2.8* in both *Corchorus* species are upregulated in drought conditions. In salinity condition, the *SnRK2.3* and 2.5 showed higher expression in jute in both root and leaf suggesting a role in the salinity tolerance. However, *SnRK2.7* and *SnRK2.8* in both species showed upregulation in the root system and downregulation in the leaf. Moreover, in saline condition, the *SnRK2.4b* expressed highly in the leaf but a lower expression in the root. In many plant species, such as *Arabidopsis*, rice, cotton, sugarbeet, and maize, it is evident that SnRK2 proteins function as transcriptional activation in ABA-signaling mechanisms in response to abiotic stresses such as salinity and drought [9]. The expression of CoSnRK2 and CcSnRK2 genes was induced by drought and salinity which may be indicative of their potential roles in stress response and fiber formation.

## Conclusion

We carried out a thorough genome-wide analysis of the SnRK2 gene family and identified and characterized seven SnRK2 genes in two jute species: *C. capsularis* and *C. olitorius*. Promoter analysis identified 188 *cis*-regulatory elements from eight major groups, including hormonal-responsive, light responsive, site-binding related, core promoter functional, stress-responsive, and ABA-responsive elements, etc. Relative expression patterns in plant tissue varied with Co- and Cc*SnRK2.7*,* Co- and CcSnRK2.8*, and Co- and Cc*SnRK2.3* in both species had higher expression in fiber than seedlings. In response to drought and salinity stresses four genes (Co- and CcSnRK2.3, Co- and CcSnRk2.5, Co- and CcSnRk2.7, and Co- and CcSnRK2.8) were upregulated, whereas two genes (Co- and CcSnRk2.6 and Co- and CcSnRK2.8) were overexpressed in waterlogging condition. This investigation provides a strong platform for future research for the functional analysis of the Co- and CcSnRK2 gene. Additionally, these findings might contribute to the genetic improvement of *Corchorus* species for the tolerance to abiotic stresses.

## Supplementary Information

**Additional file 1.** Protein sequence of AtSnRK2s, CcSnRK2s and CoSnRK2s.

**Additional file 2: Fig. S1.** Ramachandran Plot Analysis for CoSnRK2 and CcSnRK2 genes.

**Additional file 3: Table S1.** Details of RNA-Seq transcriptome data used for expression analysis of SnRK2 genes in C. capsularis and C. olitorius. **Table S2.** Identity percentage of similarity in amino acid sequence of paralogous SnRK2 genes. **Table S3.** Length of the second through the eight exons of SnRK2 genes (data as gff3 format). **Table S4.** Structural analyses of the SnRK2 proteins in both Corchorus species. **Table S5.** Gene Ontology (GO) annotation details for Co and CcSnRK2 proteins in both Corchorus species. **Table S6.** Promoter analysis of SnRK2s genes of two jute species using PlantCare database. **Table S7.** Promoter analysis of SnRK2s genes of two jutre species using PLACE database. **Table S8.** Differentially expressed SnRK2 genes in in different plant parts (a), and against waterlogging (b), Salinity (c) & Drought (d) condition. **Table S9.** Genome-wide identification and classification of SnRK2 genes in 21 plant species.

## Data Availability

All the protein sequences are available in the NCBI database.

## Abbreviations

SnRK: Sucrose non-fermenting related protein kinase
ABA: Abscisic acid
AMP: Adenosine monophosphate
AMPKs: AMP-activated protein kinases
GRAVY: Grand average of hydropathy
pI: Iso-electric point
GO: Gene Ontology
NCBI: National Center for Biotechnology Information
QC: Quality check
