# Supplementary material for: Genome-wide investigation of SnRK2 gene family in two jute species: Corchorus olitorius and Corchorus capsularis
Source: J Genet Eng Biotechnol. 2023 Jan 18;21:5. doi: 10.1186/s43141-022-00453-x (PMC9849630; doi:10.1186/s43141-022-00453-x)
Supplement: Supplementary file 1 — Additional file 1. Protein sequence of AtSnRK2s, CcSnRK2s and CoSnRK2s. [file 43141_2022_453_MOESM1_ESM.pdf]

>CcSnRK2.6
MDHRSAITVGPGMDMPIMHDSDRYELVRDIGSGNFGVARLMRDKQTEELVAVKYIERGEKIDENVQREIINHRSLRHPNIV
RFKEVILTPTHLAIVMEYASGGELFERICNAGRFSEDEARFFFQQLISGVSYCHAMQVCHRDLKLENTLLDGSPAPRLKICDFG
YSKSSVLHSQPKSTVGTPAYIAPEVLLKKEYDGKVADVWSCGVTLYVMLVGAYPFEDPEDPKNFHKTIHRILNVQYSIPDYVHI
SPECRHLISRIFVADPAKRISIPEIRNHEWFLKNLPADLMDDNTTSNQFEEADQPVQSVDEIMQIISEATIPAANTNSLNQYLT
GSLDIDDDMEEDLDSDPELDIDSSGEVIYAL

>CcSnRK2.3
MDRADLTVGPAMDMPIMHDSDRYDFVRDIGSGNFGVARLMRDKVSKELVAVKYIERGDKIDENVQREIINHRSLRHPNIV
RFKEVILTPTHLAIVMEYASGGELFERICAAGRFNEDEARFFFQQLISGVSYCHAMQVCHRDLKLENSLLDGSPAPRLKICDFG
YSKSSVLHSQPKSTVGTPAYIAPEVLLRQEYDGKIADVWSCGVTLYVMLVGAYPFEDPDEPKDFRKTIQRILTVQYSIPDFVQIS
PECLHLISRIFVADPTARITIPEIRNHEWFLKNLPADLMNENTMGNHFEEPDQPMQSTDTIMQIIAEATIPAAGAQGLNHYM
LDPLDDEDMDDLDSESELDIDSSGEIVYAM

>CcSnRK2.5
MMEERYEPLKDLGSGNFGVARLVKDKKTKELVAVKYIERGKKIDENVQREIINHRSLRHPNIIRFKEVLLTPTHLAIVMEYAAG
GELFERICSAGRFSEDEARFFFQQLISGVSYCHSMEICHRDLKLENTLLDGSPTPRLKICDFGYSKSAVLHSQPKSTVGTPAYIAP
EVLSRKEYDGKVADVWSCGVTLYVMLVGAYPFEDPEDPRNFRKTIGRIMSVQYSIPDYVRVSADCRQLLSRIFVPNPAKRITIP
EIKQHPWFLKNLPKELVEIEKTNFSESNRDQPSQSVEEIMRIIQEAKTPAAGAKVGEQAVAGSSEPDDADAEADLESEIDVSG
EFDATA

>CcSnRK2.4a
MEKYEVVKDLGAGNFGVARLLRHKETKELVAMKYIERGHKVVLTPTHLAIVMEYAAGGELFERICSAGRFSEDEARYFFQQLI
SGVNYCHSMQICHRDLKLENTLLDGSPAPRLKICDFGYSKSSLLHSRPKSTVGTPAYIAPEVLSRREYDGKCMTILVNGRAYPF
EDQEDPRNFRKTIQRIMAVQYKIPDYVHISQDCRNLLSRIFVANSSRRITLKEIKNHPWFLKNLPRELTDAAQASYYRRDNPTF
SLQTVEDIMKIVEEARSPPPASVPVKGFGWGQDDDEEEDVEADVEEEDQEDEYDKRVKEVHASGEFQIH

>CcSnRK2.4b
MEKYEVVKDLGAGNFGVARLLRNKETKELVAMKYIERGHKVVLTPTHLAIVMEYAAGGELFERICSAGRFSEDEARYFFQQLI
SGVNYCHSMQICHRDLKLENTLLDGSPAPHLKICDFGYSKSSLLHSRPKSTVGTPAYIAPEVLSRREYDGKGALVGQAEIECLL
NKLDVWSCGVTLYVMLVGAYPFEDQEDPRNFRKTIQRIMAVQYKIPDYVHISQDCRNLLSRIFVANSSRRITLKEIKNHPWFL
KNLPRELTDAAQASYYRRDNPTFSLQSVEDIMKIVEEARSPPPASVPVKGFSWGQDDDEEEDVEAEVEEEDEEDEYDKRVKE
VHASGEFQIH

>CcSnRK2.8
KANYCCPQQQDTENVDVWMMQTDVLLTPTHLAIVMEYAAGGELFERICNAGRFSEDEARFFFQQLISGVSYCHSMQICHR
DLKLENTLLDGSIAPRVKICDFGYSKSSVFHSQPKSTVGTPAYIAPEVLSKKEYDGKIADVWSCGVTLYVMLVGAYPFEDPDDP
KNFRKTIGRILSVHYSIPDYVRVSIECKHLLSRIFVANPEKRITIPEIKSHPWFLKNLPIELMEGGSWQSHDVNNPSQSLEEVQSII
QEAMKSTEVAKNNGGLFMEGSMDLDDFDADADLEDIETSGDFVCQL

>CcSnRK2.7
MERYEILKDIGSGNFGVAKLVRDKWSGELYAVKYIERGPKIDEHVQREIMNHRSLKHPNIIRFKEVFLTPTHLAIVMEYAAGGE
LFERICTAGRFSEDEARFFFQQLISGVSYCHAMSSVFHSQPKSTVGTPAYIAPEVLSRKEYDGKIADVWSCGVTLYVMLVGAY
PFEDPEDPRNFRKTIQRILSVHYSIPDYVRISKECRHLLSRIFVANPEKRITIPEIKQHPWFLKNLPIEFMEEENGNLETDQENDN
DQSQQSIEEILSIVDEARKPGEEGPKVGSQFLGGSMDLDDLDDADIDDIETSGDFVCALQV

>CoSnRK2.6
MDHRSAITVGPGMDMPIMHDSDRYELVRDIGSGNFGVARLMRDKQTEELVAVKYIERGEKIDENVQREIINHRSLRHPNIV
RFKEVILTPTHLAIVMEYASGGELFERICNAGRFSEDEARFFFQQLISGVSYCHAMQVCHRDLKLENTLLDGSPAPRLKICDFG
YSKSSVLHSQPKSTVGTPAYIAPEVLLKKEYDGKVADVWSCGVTLYVMLVGAYPFEDPEDPKNFHKTIHRILNVQYSIPDYVHI
SPECRHLISRIFVADPAKRISIPEIRNHEWFLKNLPADLMDDNTTSNQFEEADQPVQSVDEIMQIISEATIPAANTNSLNQYLT
GSLDIDDDMEEDLDSDPELDIDSSGEVIYAL

>CoSnRK2.3
MDRADLTVGPAMDMPIMHDSDRYDFVRDIGSGNFGVARLMRDKVSKELVAVKYIERGDKIDENVQREIINHRSLRHPNIV
RFKEVILTPTHLAIVMEYASGGELFERICAAGRFNEDEARFFFQQLISGVSYCHAMQVCHRDLKLENTLLDGSPAPRLKICDFG
YSKSTVGTPAYIAPEVLLRQEYDGKIADVWSCGVTLYVMLVGAYPFEDPDEPKDFRKTIQRILTVQYSIPDFVQISPECRHLISRI
FVADPTARITIPEIRNHEWFLKNLPADLMNENTMGNHFEEPDQPMQSTDTIMQIIAEATIPAAGAQGLNHYMLDPLDDED
MDDLDSESELDIDSSGEIVYAM

>CoSnRK2.5
MMEERYEPLKDLGSGNFGVARLVKDKKTKELVAVKYIERGKKIDENVQREIINHRSLRHPNIIRFKEVLLTPTHLAIVMEYAAG
GELFERICSAGRFSEDEARFFFQQLISGVSYCHSMEICHRDLKLENTLLDGSPTPRLKICDFGYSKSAVLHSQPKSTVGTPAYIAP
EVLSRKEYDGKVADVWSCGVTLYVMLVGAYPFEDPEDPRNFRKTIGRIMSVQYSIPDYVRVSADCRQLLSRIFVSNPAKRITIP
EIKQHPWFLKNLPKELVEIEKTNFAESNRDQPSQSVEEIMRIIQEAKTPAAGAKVGEQAVAGSSEPDDADAEADLESEIDVSG
EFDAPA

>CoSnRK2.8
MERYEIVKDIGSGNFGVAKLVRDKWTKELFAVKFIERGQKIDEHVQREIMNHRSLKHPNIVRFKEVLLTPTHLAIVMEYAAGG
ELFERICNAGRFSEDEARFFFQQLISGVSYCHSMQICHRDLKLENTLLDGSIAPRVKICDFGYSKSSVFHSQPKSTVGTPAYIAPE
VLSKKEYDGKIADVWSCGVTLYVMLVGAYPFEDPDDPKNFRKTIGRILSVHYSIPDYVRVSIECKHLLSRIFVANPEKRITIPEIKS
HPWFLKNLPIELMEGGSWQSHDVNNPSQSLEEVQSIIQEAMKSTEVAKNNGGLFMEGSMDLDDFDADDADLEDIETSGD
FVCQL

>CoSnRK2.4b
MEKYELVKDIGSGNFGVARLMRNKETKELVAMKYIERGHKIDENVAREIINHRSLRHPNIIRFKEVVLTPTHLAIVMEYAAGG
ELFERICNAGRFSEDEQICHRDLKLENTLLDGSPAPRLKICDFGYSKSSLLHSRPKSTVGTPAYIAPEVLSRREYDGKMADVWSC
GVTLYVMLVGAYPFEDQEDPKNFRKTISRIMSVQYKIPDYVHISQDCRHLLSRIFVASPSRRISIKDIKSHPWFLKNLPRELTEA
AQAVYYRKENPTFSLQTVEEIMKIVEEAKAAPPVSRSIGGFGWGGEEDGDLKEEDVEEEEEEDEYEKTVKEVHASGEVHIS

>CoSnRK2.4a
MEKYEVVKDLGAGNFGVARLLRHKETKELVAMKYIERGHKVVLTPTHLAIVMEYAAGGELFERICSAGRFSEDEARYFFQQLI
SGVNYCHSMQICHRDLKLENTLLDGSPAPRLKICDFGYSKSSLLHSRPKSTVGTPAYIAPEVLSRREYDGKCMTILVNGRTLVL
GDSEGAYPFEDQEDPRNFRKTIQRIMAVQYKIPDYVHISQDCRNLLSRIFVANSSRRITLKEIKNHPWFLKNLPRELTDAAQAS
YYRRDNPTFSLQTVEDIMKIVEEARSPPPASVPVKGFGWGQDDDEEEDVEADVEEEDQEDEYDKRVKEVHASGEFQIH

>CoSnRK2.7
MERYEILKDIGSGNFGVAKLVRDKWSGELYAVKYIERGPKIDEHVQREIMNHRSLKHPNIIRFKEVFLTPTHLAIVMEYAAGGE
LFERICTAGRFSEDEARFFFQQLISGVSYCHAMSSVFHSQPKSTVGTPAYIAPEVLSRKEYDGKIADVWSCGVTLYVMLVGAY
PFEDPEDPRNFRKTIQRILSVHYSIPDYVRISKECRHLLSRIFVANPEKRITIPEIKQHPWFLKNLPIEFMEEENGNLETDQENDD
QSQQSIEEILSIVDEARKPGEEGPKVGSQFLGGSMDLDDLDDADIDDIETSGDFVCALQV

>AtSnRK2.1
MDKYDVVKDLGAGNFGVARLLRHKDTKELVAMKYIERGRKIDENVAREIINHRSLKHPNIIRFKEVILTPTHLAIVMEYASGGE
LFDRICTAGRFSEAEARYFFQQLICGVDYCHSLQICHRDLKLENTLLDGSPAPLLKICDFGYSKSSILHSRPKSTVGTPAYIAPEVL
SRREYDGKHADVWSCGVTLYVMLVGAYPFEDPNDPKNFRKTIQRIMAVQYKIPDYVHISQECKHLLSRIFVTNSAKRITLKEIK
NHPWYLKNLPKELLESAQAAYYKRDTSFSLQSVEDIMKIVGEARNPAPSTSAVKSSGSGADEEEEEDVEAEVEEEEDDEDEYE
KHVKEAQSCQESDKA

>AtSnRK2.2
MDPATNSPIMPIDLPIMHDSDRYDFVKDIGSGNFGVARLMTDRVTKELVAVKYIERGEKIDENVQREIINHRSLRHPNIVRFK
EVILTPSHLAIVMEYAAGGELYERICNAGRFSEDEARFFFQQLISGVSYCHAMQICHRDLKLENTLLDGSPAPRLKICDFGYSKS
SVLHSQPKSTVGTPAYIAPEILLRQEYDGKLADVWSCGVTLYVMLVGAYPFEDPQEPRDYRKTIQRILSVTYSIPEDLHLSPECR
HLISRIFVADPATRITIPEITSDKWFLKNLPGDLMDENRMGSQFQEPEQPMQSLDTIMQIISEATIPTVRNRCLDDFMADNLD
LDDDMDDFDSESEIDVDSSGEIVYAL

>AtSnRK2.3
MDRAPVTTGPLDMPIMHDSDRYDFVKDIGSGNFGVARLMRDKLTKELVAVKYIERGDKIDENVQREIINHRSLRHPNIVRFK
EVILTPTHLAIIMEYASGGELYERICNAGRFSEDEARFFFQQLLSGVSYCHSMQICHRDLKLENTLLDGSPAPRLKICDFGYSKSS
VLHSQPKSTVGTPAYIAPEVLLRQEYDGKIADVWSCGVTLYVMLVGAYPFEDPEEPRDYRKTIQRILSVKYSIPDDIRISPECCH
LISRIFVADPATRISIPEIKTHSWFLKNLPADLMNESNTGSQFQEPEQPMQSLDTIMQIISEATIPAVRNRCLDDFMTDNLDLD
DDMDDFDSESEIDIDSSGEIVYAL

>AtSnRK2.4
MDKYELVKDIGAGNFGVARLMKVKNSKELVAMKYIERGPKIDENVAREIINHRSLRHPNIIRFKEVVLTPTHLAIAMEYAAGG
ELFERICSAGRFSEDEARYFFQQLISGVSYCHAMQICHRDLKLENTLLDGSPAPRLKICDFGYSKSSLLHSRPKSTVGTPAYIAPE
VLSRREYDGKMADVWSCGVTLYVMLVGAYPFEDQEDPKNFRKTIQKIMAVQYKIPDYVHISQDCKNLLSRIFVANSLKRITIA
EIKKHSWFLKNLPRELTETAQAAYFKKENPTFSLQTVEEIMKIVADAKTPPPVSRSIGGFGWGGNGDADGKEEDAEDVEEEE
EEVEEEEEDDEDEYDKTVKEVHASGEVRIS

>AtSnRK2.5
MDKYEVVKDLGAGNFGVARLLRHKETKELVAMKYIERGRKIDENVAREIINHRSLRHPNIIRFKEVILTPTHLAIVMEYASGGE
LFERICNAGRFSEAEARYFFQQLICGVDYCHSLQICHRDLKLENTLLDGSPAPLLKICDFGYSKSSLLHSRPKSTVGTPAYIAPEVL
SRREYDGKHADVWSCGVTLYVMLVGGYPFEDPDDPRNFRKTIQRIMAVQYKIPDYVHISQECRHLLSRIFVTNSAKRITLKEIK
KHPWYLKNLPKELTEPAQAAYYKRETPSFSLQSVEDIMKIVGEARNPAPSSNAVKGFDDDEEDVEDEVEEEEEEEEEEEEEEE
EEEDEYEKHVKEAHSCQEPPKA

>AtSnRK2.6
MDRPAVSGPMDLPIMHDSDRYELVKDIGSGNFGVARLMRDKQSNELVAVKYIERGEKIDENVKREIINHRSLRHPNIVRFKE
VILTPTHLAIVMEYASGGELFERICNAGRFSEDEARFFFQQLISGVSYCHAMQVCHRDLKLENTLLDGSPAPRLKICDFGYSKSS
VLHSQPKSTVGTPAYIAPEVLLKKEYDGKVADVWSCGVTLYVMLVGAYPFEDPEEPKNFRKTIHRILNVQYAIPDYVHISPECR
HLISRIFVADPAKRISIPEIRNHEWFLKNLPADLMNDNTMTTQFDESDQPGQSIEEIMQIIAEATVPPAGTQNLNHYLTGSLDI
DDDMEEDLESDLDDLDIDSSGEIVYAM

>AtSnRK2.7
MERYDILRDLGSGNFGVAKLVREKANGEFYAVKYIERGLKIDEHVQREIINHRDLKHPNIIRFKEVFVTPTHLAIVMEYAAGGE
LFERICNAGRFSEDEGRYYFKQLISGVSYCHAMQICHRDLKLENTLLDGSPSSHLKICDFGYSKSSVLHSQPKSTVGTPAYVAPE
VLSRKEYNGKIADVWSCGVTLYVMLVGAYPFEDPEDPRNIRNTIQRILSVHYTIPDYVRISSECKHLLSRIFVADPDKRITVPEIE
KHPWFLKGPLVVPPEEEKCDNGVEEEEEEEKCRQSVEEIVKIIEEARKGVNGTDNNGGLGLIDGSIDLDDIDDADIYDDVDD
DEERNGDFVCAL

>AtSnRK2.8
MERYEIVKDIGSGNFGVAKLVRDKFSKELFAVKFIERGQKIDEHVQREIMNHRSLIHPNIIRFKEVLLTATHLALVMEYAAGGE
LFGRICSAGRFSEDEARFFFQQLISGVNYCHSLQICHRDLKLENTLLDGSEAPRVKICDFGYSKSGVLHSQPKTTVGTPAYIAPE
VLSTKEYDGKIADVWSCGVTLYVMLVGAYPFEDPSDPKDFRKTIGRILKAQYAIPDYVRVSDECRHLLSRIFVANPEKRITIEEIK
NHSWFLKNLPVEMYEGSLMMNGPSTQTVEEIVWIIEEARKPITVATGLAGAGGSGGSSNGAIGSSSMDLDDLDTDFDDIDT
ADLLSPL

>AtSnRK2.9
MEKYEMVKDLGFGNFGLARLMRNKQTNELVAVKFIDRGYKIDENVAREIINHRALNHPNIVRFKEVVLTPTHLGIVMEYAAG
GELFERISSVGRFSEAEARYFFQQLICGVHYLHALQICHRDLKLENTLLDGSPAPRLKICDFGYSKSSVLHSNPKSTVGTPAYIAP
EVFCRSEYDGKSVDVWSCGVALYVMLVGAYPFEDPKDPRNFRKTVQKIMAVNYKIPGYVHISEDCRKLLSRIFVANPLHRSTL
KEIKSHAWFLKNLPRELKEPAQAIYYQRNVNLINFSPQRVEEIMKIVGEARTIPNLSRPVESLGSDKKDDDEEEYLDANDEEWY
DDYA

>AtSnRK2.10
MDKYELVKDIGAGNFGVARLMRVKNSKELVAMKYIERGPKIDENVAREIINHRSLRHPNIIRFKEVVLTPTHIAIAMEYAAGG
ELFERICSAGRFSEDEARYFFQQLISGVSYCHAMQICHRDLKLENTLLDGSPAPRLKICDFGYSKSSLLHSMPKSTVGTPAYIAP
EVLSRGEYDGKMADVWSCGVTLYVMLVGAYPFEDQEDPKNFKKTIQRIMAVKYKIPDYVHISQDCKHLLSRIFVTNSNKRITI
GDIKKHPWFLKNLPRELTEIAQAAYFRKENPTFSLQSVEEIMKIVEEAKTPARVSRSIGAFGWGGGEDAEGKEEDAEEEVEEV
EEEEDEEDEYDKTVKQVHASMGEVRVS
