# Supplementary material for: Genome-wide investigation of SnRK2 gene family in two jute species: Corchorus olitorius and Corchorus capsularis
Source: J Genet Eng Biotechnol. 2023 Jan 18;21:5. doi: 10.1186/s43141-022-00453-x (PMC9849630; doi:10.1186/s43141-022-00453-x)
Supplement: Supplementary file 2 — Additional file 2: Fig. S1. Ramachandran Plot Analysis for CoSnRK2 and CcSnRK2 genes. [file 43141_2022_453_MOESM2_ESM.pdf]

# Ramachandran Plot

**CoSnRK2.3**

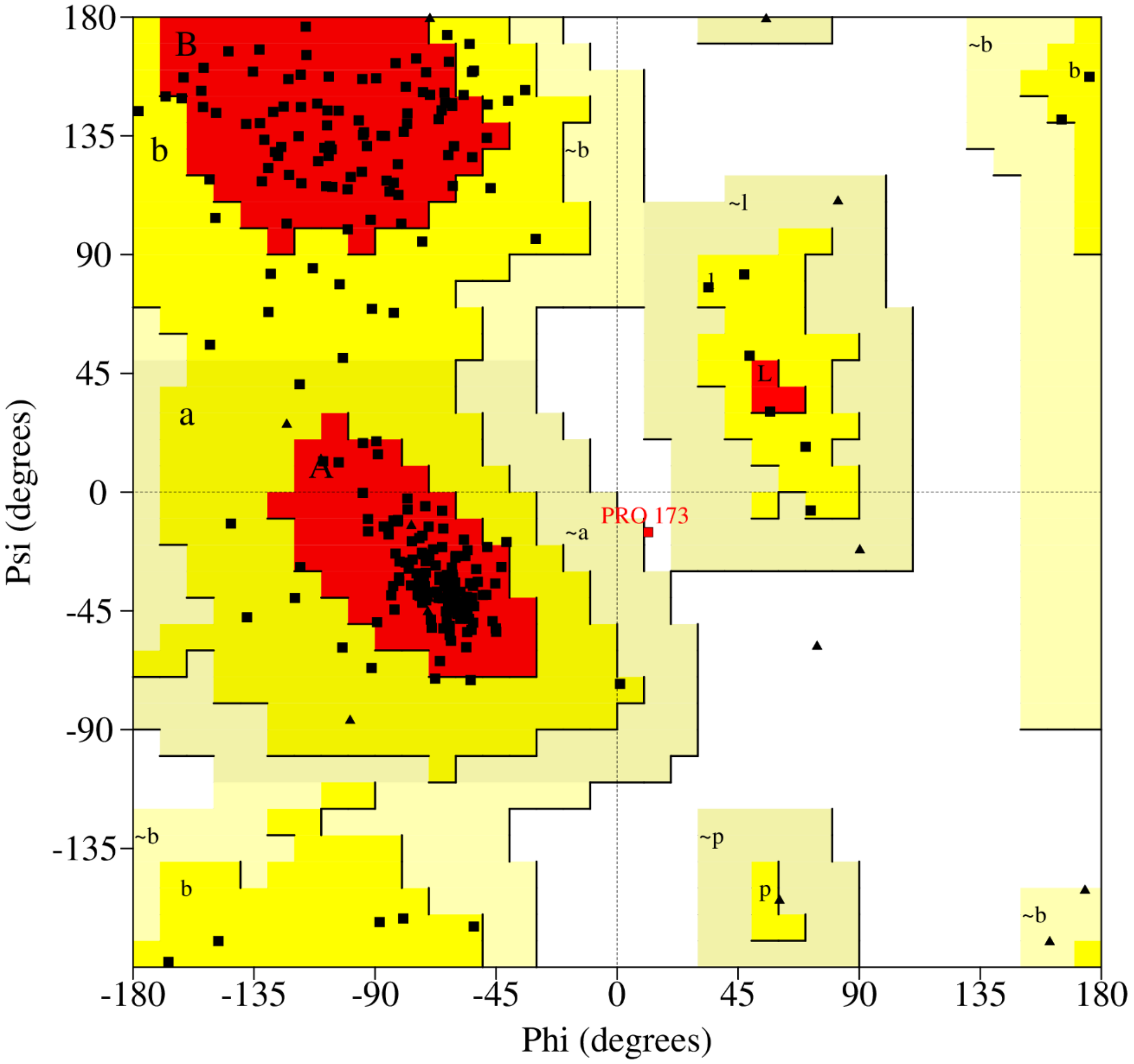


Plot statistics

| | | |
|---|---|---|
| Residues in most favoured regions [A,B,L] | 202 | 82.8% |
| Residues in additional allowed regions [a,b,l,p] | 42 | 17.2% |
| Residues in generously allowed regions [~a,~b,~l,~p] | 0 | 0.0% |
| Residues in disallowed regions | 0 | 0.0% |
| | ---- | ------ |
| Number of non-glycine and non-proline residues | 244 | 100.0% |
| Number of end-residues (excl. Gly and Pro) | 3 | |
| Number of glycine residues (shown as triangles) | 15 | |
| Number of proline residues | 16 | |
| | ---- | |
| Total number of residues | 278 | |

Based on an analysis of 118 structures of resolution of at least 2.0 Angstroms and R-factor no greater than 20%, a good quality model would be expected to have over 90% in the most favoured regions.

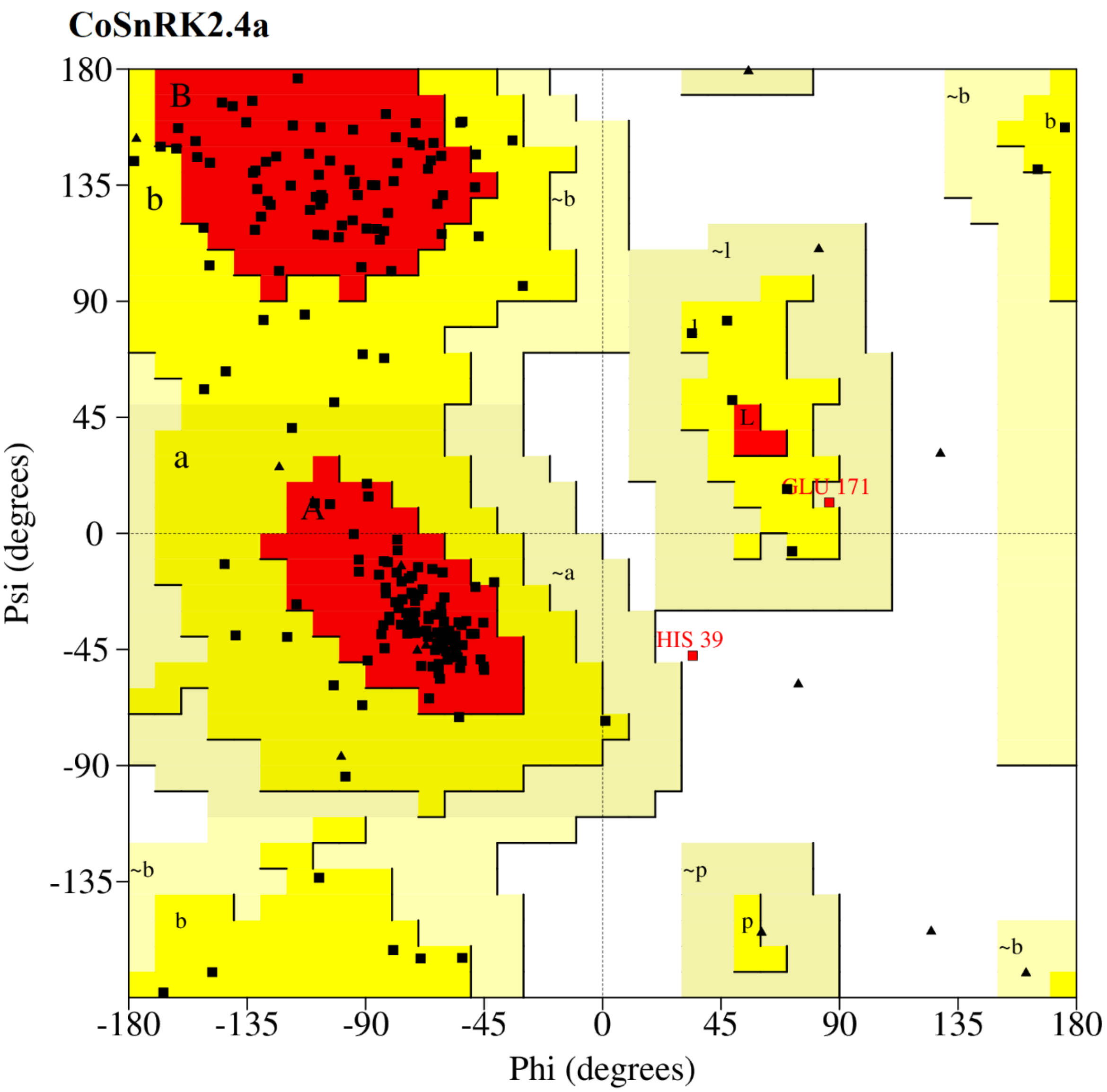


Plot statistics

| | | |
|---|---|---|
| Residues in most favoured regions [A,B,L] | 160 | 80.4% |
| Residues in additional allowed regions [a,b,l,p] | 37 | 18.6% |
| Residues in generously allowed regions [~a,~b,~l,~p] | 1 | 0.5% |
| Residues in disallowed regions | 1 | 0.5% |
| | ---- | ------ |
| Number of non-glycine and non-proline residues | 199 | 100.0% |
| Number of end-residues (excl. Gly and Pro) | 3 | |
| Number of glycine residues (shown as triangles) | 14 | |
| Number of proline residues | 10 | |
| | ---- | |
| Total number of residues | 226 | |

Based on an analysis of 118 structures of resolution of at least 2.0 Angstroms and R-factor no greater than 20%, a good quality model would be expected to have over 90% in the most favoured regions.

## CoSnRK2.4b

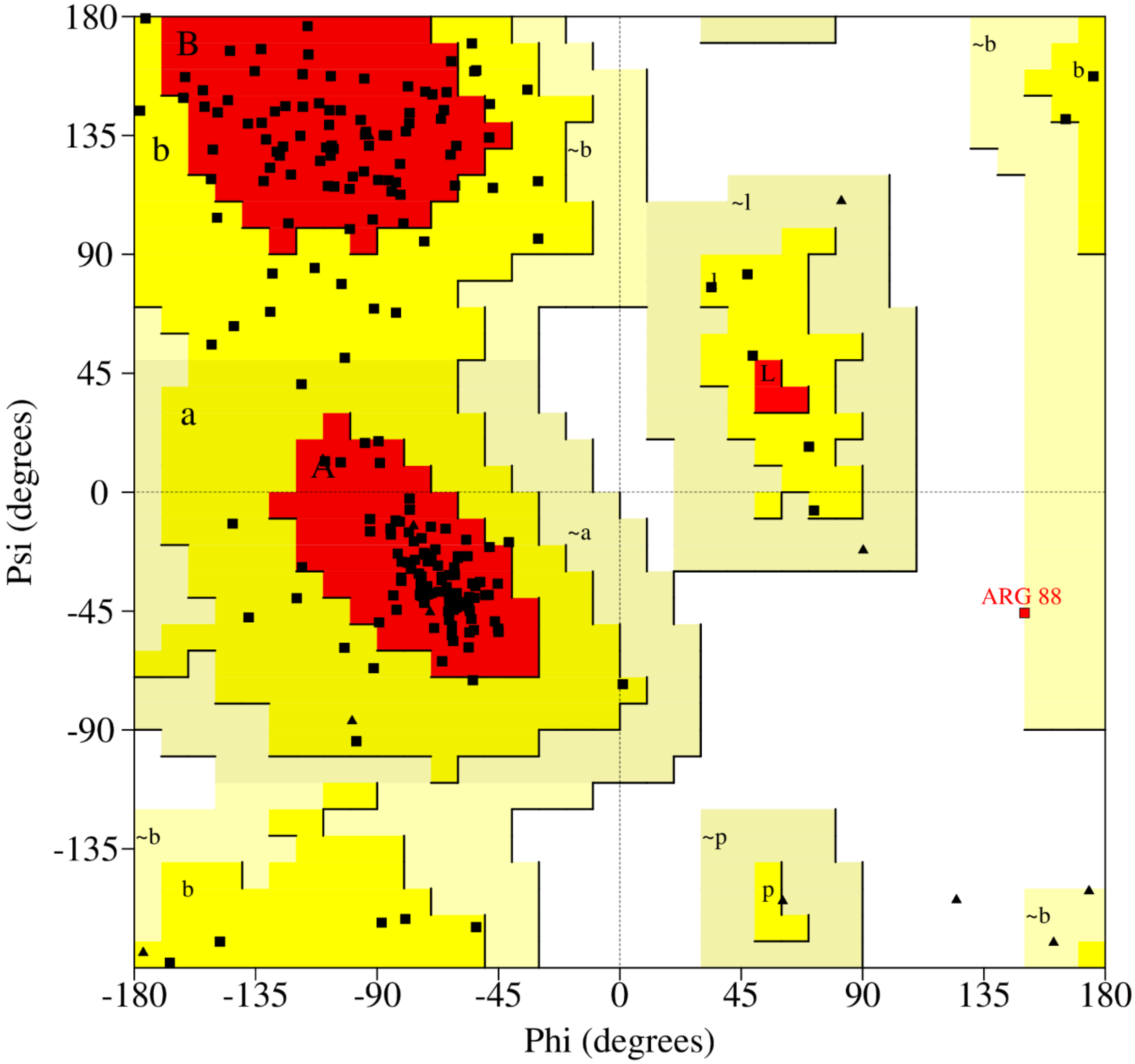


Plot statistics

| | | |
|---|---|---|
| Residues in most favoured regions [A,B,L] | 162 | 79.0% |
| Residues in additional allowed regions [a,b,l,p] | 42 | 20.5% |
| Residues in generously allowed regions [~a,~b,~l,~p] | 1 | 0.5% |
| Residues in disallowed regions | 0 | 0.0% |
| | ---- | ------ |
| Number of non-glycine and non-proline residues | 205 | 100.0% |
| Number of end-residues (excl. Gly and Pro) | 3 | |
| Number of glycine residues (shown as triangles) | 12 | |
| Number of proline residues | 12 | |
| | ---- | |
| Total number of residues | 232 | |

Based on an analysis of 118 structures of resolution of at least 2.0 Angstroms and R-factor no greater than 20%, a good quality model would be expected to have over 90% in the most favoured regions.

**CoSnRK2.5**

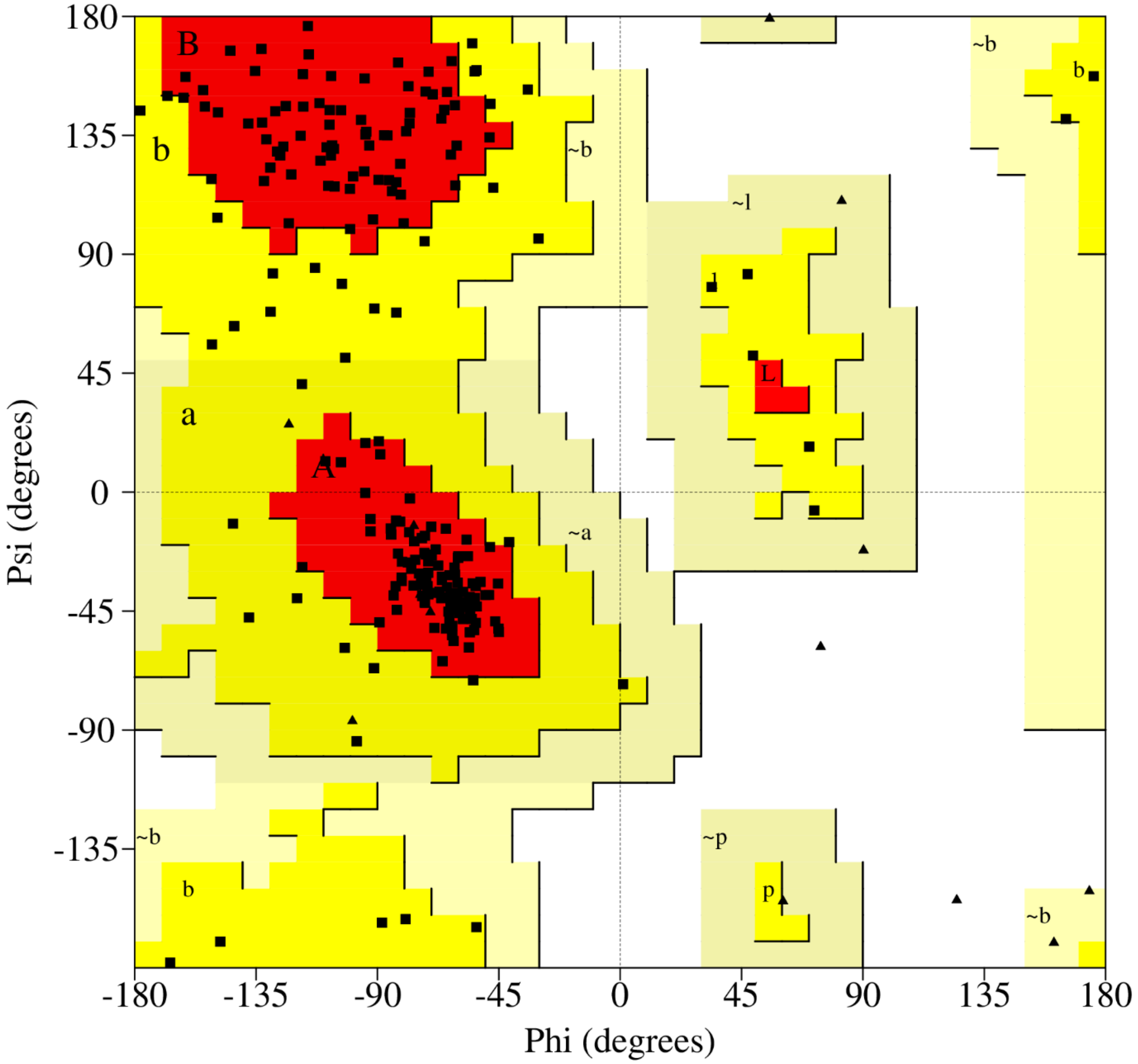


Plot statistics

| | | |
|---|---|---|
| Residues in most favoured regions [A,B,L] | 176 | 81.1% |
| Residues in additional allowed regions [a,b,l,p] | 41 | 18.9% |
| Residues in generously allowed regions [~a,~b,~l,~p] | 0 | 0.0% |
| Residues in disallowed regions | 0 | 0.0% |
| | ---- | ------ |
| Number of non-glycine and non-proline residues | 217 | 100.0% |
| Number of end-residues (excl. Gly and Pro) | 3 | |
| Number of glycine residues (shown as triangles) | 14 | |
| Number of proline residues | 15 | |
| | ---- | |
| Total number of residues | 249 | |

Based on an analysis of 118 structures of resolution of at least 2.0 Angstroms and R-factor no greater than 20%, a good quality model would be expected to have over 90% in the most favoured regions.

**CoSnRK2.6**

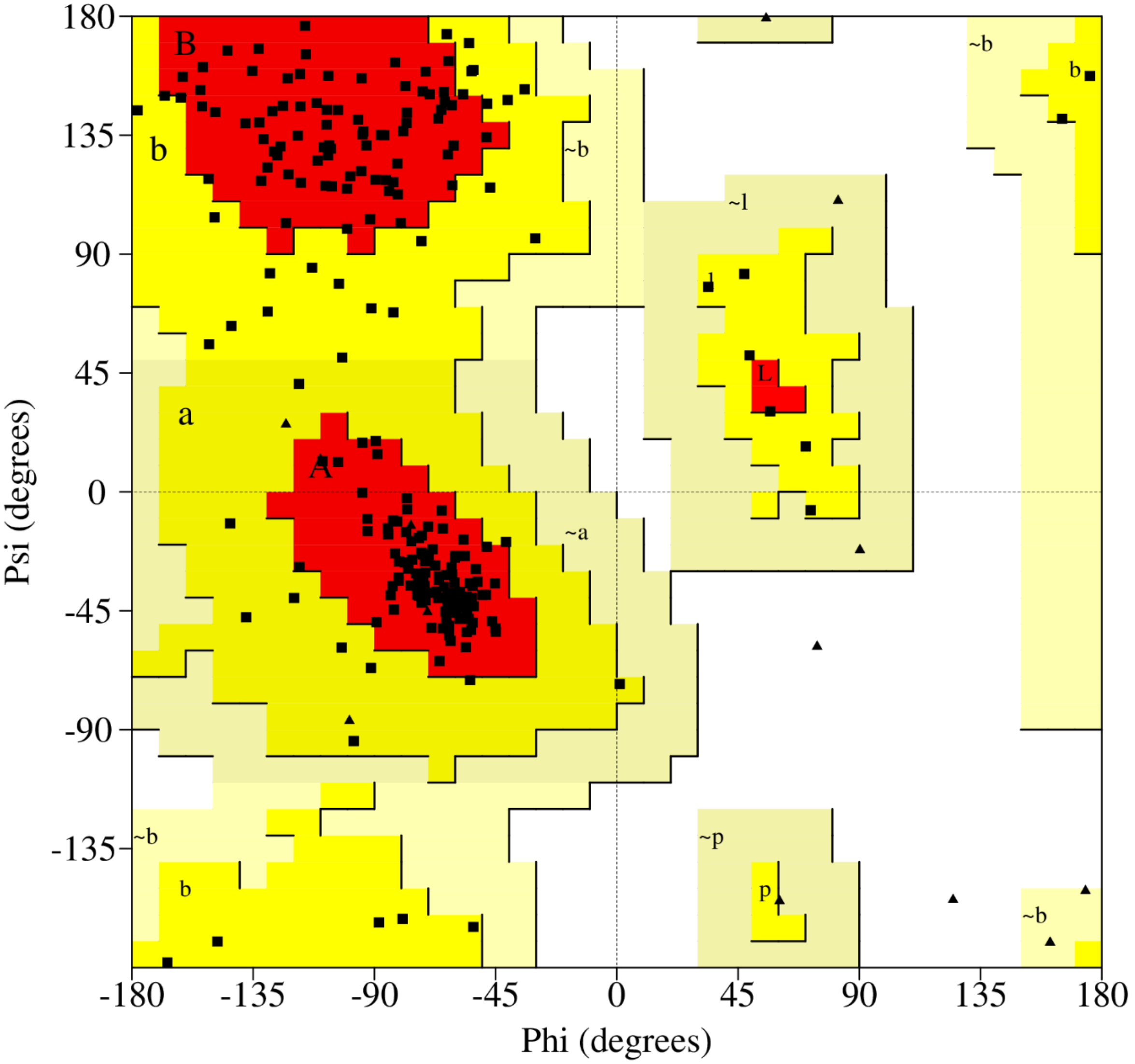


Plot statistics

| | | |
|---|---|---|
| Residues in most favoured regions [A,B,L] | 186 | 81.2% |
| Residues in additional allowed regions [a,b,l,p] | 43 | 18.8% |
| Residues in generously allowed regions [~a,~b,~l,~p] | 0 | 0.0% |
| Residues in disallowed regions | 0 | 0.0% |
| | ---- | ------ |
| Number of non-glycine and non-proline residues | 229 | 100.0% |
| Number of end-residues (excl. Gly and Pro) | 6 | |
| Number of glycine residues (shown as triangles) | 13 | |
| Number of proline residues | 15 | |
| | ---- | |
| Total number of residues | 263 | |

Based on an analysis of 118 structures of resolution of at least 2.0 Angstroms and R-factor no greater than 20%, a good quality model would be expected to have over 90% in the most favoured regions.

# CoSnRK2.7

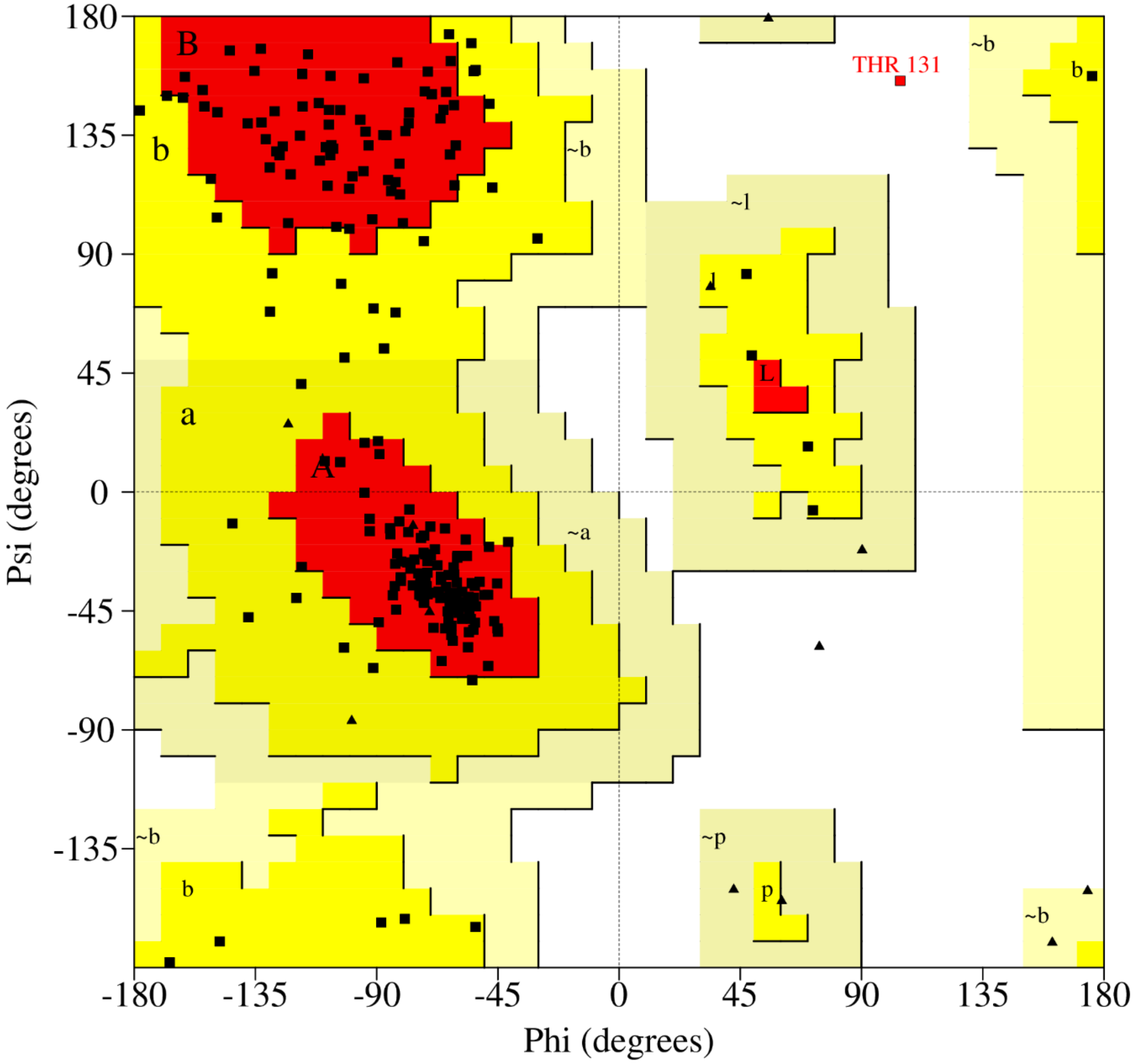


Plot statistics

| | | |
|---|---|---|
| Residues in most favoured regions [A,B,L] | 170 | 82.5% |
| Residues in additional allowed regions [a,b,l,p] | 35 | 17.0% |
| Residues in generously allowed regions [~a,~b,~l,~p] | 0 | 0.0% |
| Residues in disallowed regions | 1 | 0.5% |
| | ---- | ------ |
| Number of non-glycine and non-proline residues | 206 | 100.0% |
| Number of end-residues (excl. Gly and Pro) | 3 | |
| Number of glycine residues (shown as triangles) | 13 | |
| Number of proline residues | 14 | |
| | ---- | |
| Total number of residues | 236 | |

Based on an analysis of 118 structures of resolution of at least 2.0 Angstroms and R-factor no greater than 20%, a good quality model would be expected to have over 90% in the most favoured regions.

**CoSnRK2.8**

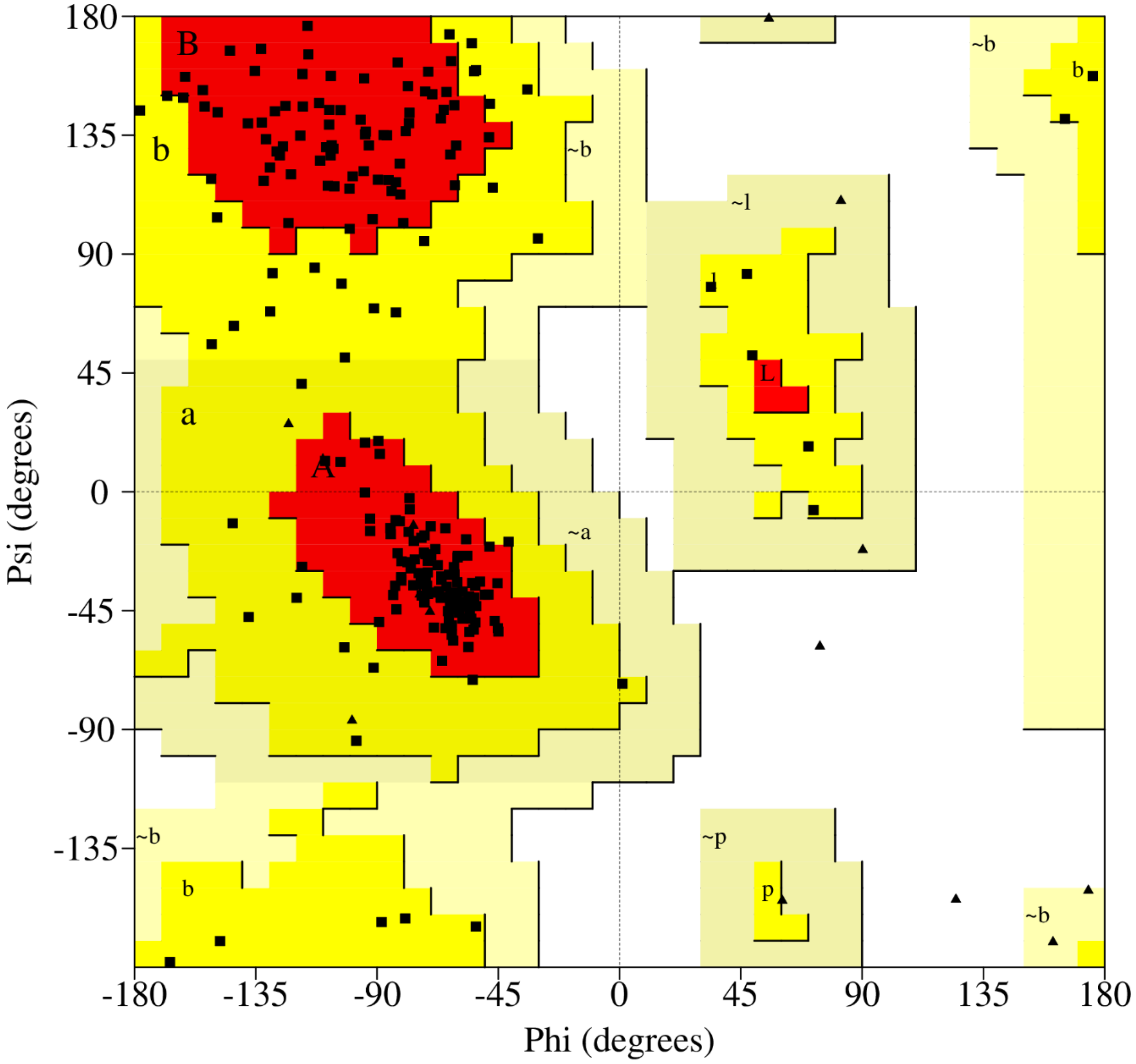


Plot statistics

| | | |
|---|---|---|
| Residues in most favoured regions [A,B,L] | 178 | 80.9% |
| Residues in additional allowed regions [a,b,l,p] | 42 | 19.1% |
| Residues in generously allowed regions [~a,~b,~l,~p] | 0 | 0.0% |
| Residues in disallowed regions | 0 | 0.0% |
| | ---- | ------ |
| Number of non-glycine and non-proline residues | 220 | 100.0% |
| Number of end-residues (excl. Gly and Pro) | 5 | |
| Number of glycine residues (shown as triangles) | 14 | |
| Number of proline residues | 13 | |
| | ---- | |
| Total number of residues | 252 | |

Based on an analysis of 118 structures of resolution of at least 2.0 Angstroms
and R-factor no greater than 20%, a good quality model would be expected
to have over 90% in the most favoured regions.

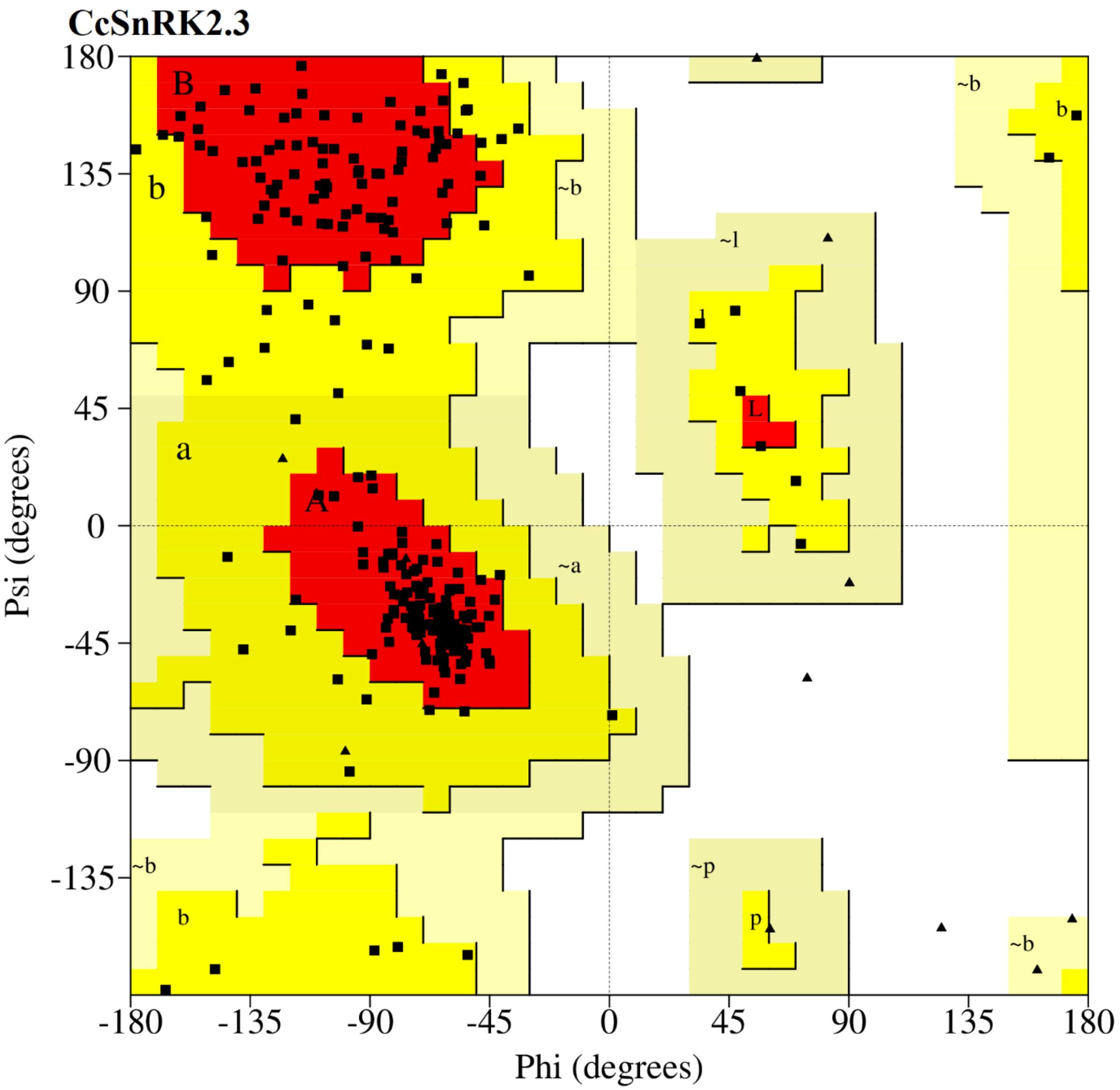


Plot statistics

| | | |
|---|---|---|
| Residues in most favoured regions [A,B,L] | 193 | 81.4% |
| Residues in additional allowed regions [a,b,l,p] | 44 | 18.6% |
| Residues in generously allowed regions [~a,~b,~l,~p] | 0 | 0.0% |
| Residues in disallowed regions | 0 | 0.0% |
| | ---- | ------ |
| Number of non-glycine and non-proline residues | 237 | 100.0% |
| Number of end-residues (excl. Gly and Pro) | 5 | |
| Number of glycine residues (shown as triangles) | 14 | |
| Number of proline residues | 16 | |
| | ---- | |
| Total number of residues | 272 | |

Based on an analysis of 118 structures of resolution of at least 2.0 Angstroms and R-factor no greater than 20%, a good quality model would be expected to have over 90% in the most favoured regions.

**CcSnRK2.4a**

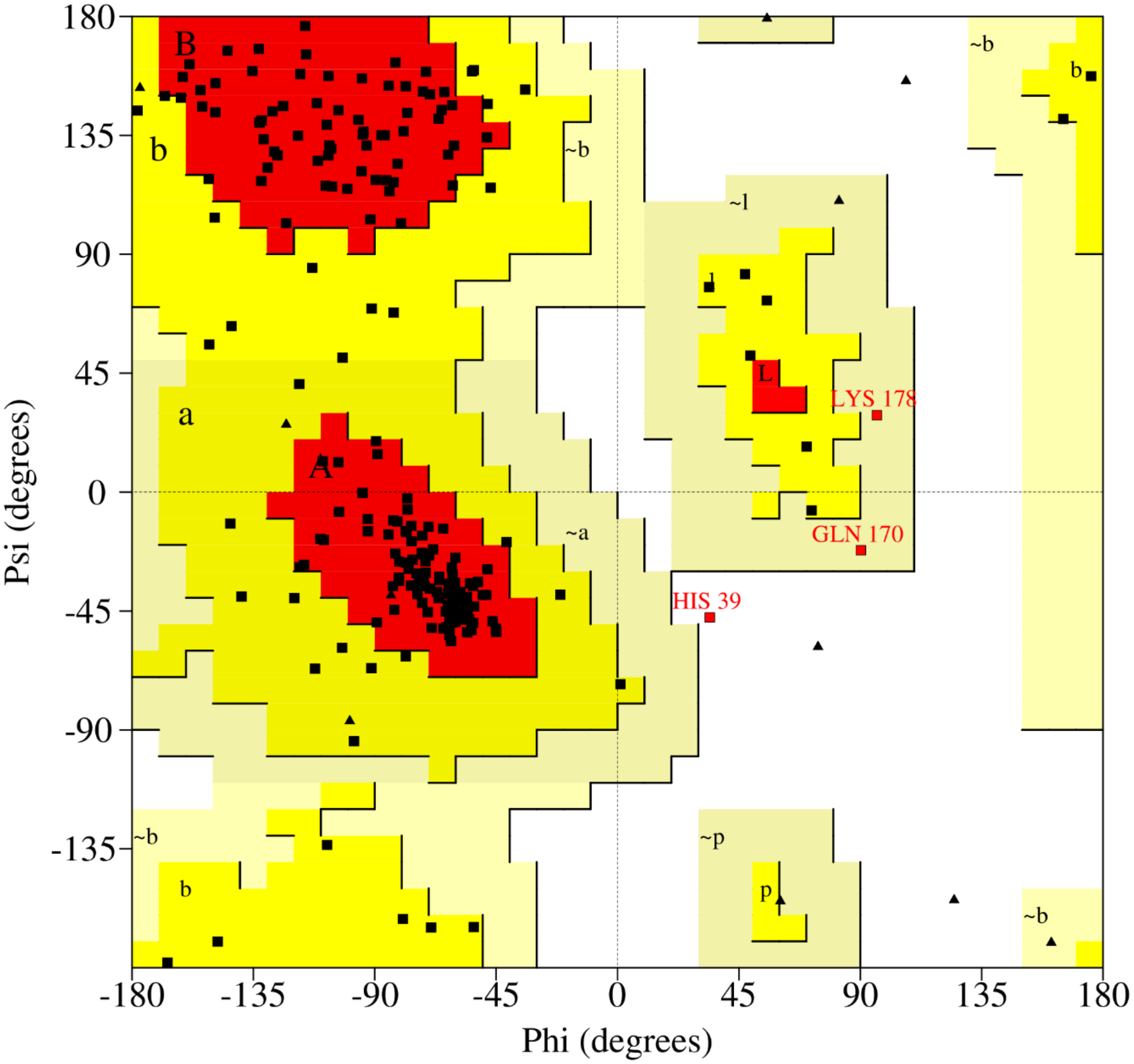


Plot statistics

| | | |
|---|---|---|
| Residues in most favoured regions [A,B,L] | 166 | 80.2% |
| Residues in additional allowed regions [a,b,l,p] | 38 | 18.4% |
| Residues in generously allowed regions [~a,~b,~l,~p] | 2 | 1.0% |
| Residues in disallowed regions | 1 | 0.5% |
| | ---- | ------ |
| Number of non-glycine and non-proline residues | 207 | 100.0% |
| Number of end-residues (excl. Gly and Pro) | 1 | |
| Number of glycine residues (shown as triangles) | 13 | |
| Number of proline residues | 11 | |
| | ---- | |
| Total number of residues | 232 | |

Based on an analysis of 118 structures of resolution of at least 2.0 Angstroms and R-factor no greater than 20%, a good quality model would be expected to have over 90% in the most favoured regions.

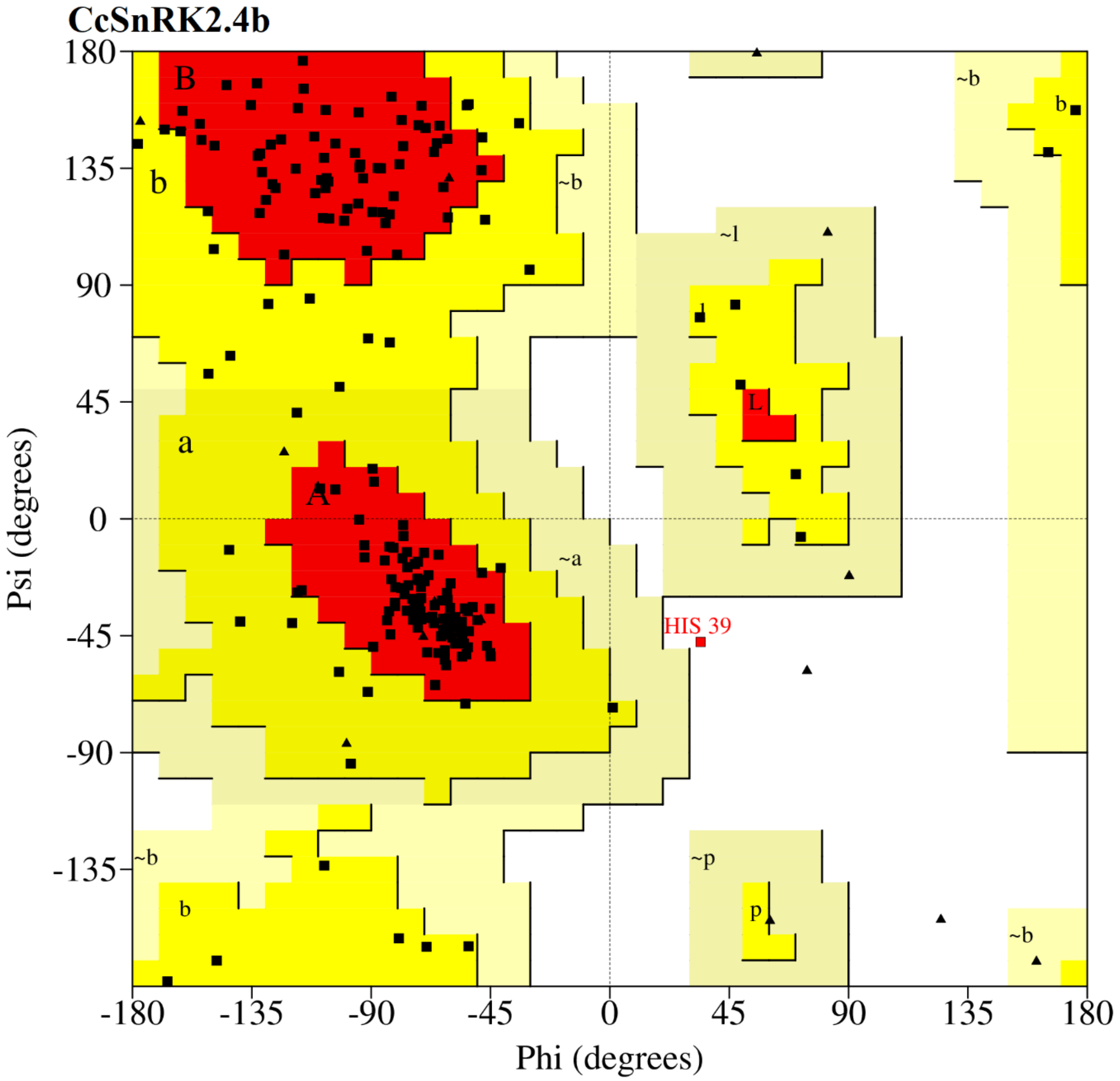


Plot statistics

| | | |
|---|---|---|
| Residues in most favoured regions [A,B,L] | 161 | 80.9% |
| Residues in additional allowed regions [a,b,l,p] | 37 | 18.6% |
| Residues in generously allowed regions [~a,~b,~l,~p] | 0 | 0.0% |
| Residues in disallowed regions | 1 | 0.5% |
| | ---- | ------ |
| Number of non-glycine and non-proline residues | 199 | 100.0% |
| Number of end-residues (excl. Gly and Pro) | 3 | |
| Number of glycine residues (shown as triangles) | 15 | |
| Number of proline residues | 8 | |
| | ---- | |
| Total number of residues | 225 | |

Based on an analysis of 118 structures of resolution of at least 2.0 Angstroms and R-factor no greater than 20%, a good quality model would be expected to have over 90% in the most favoured regions.

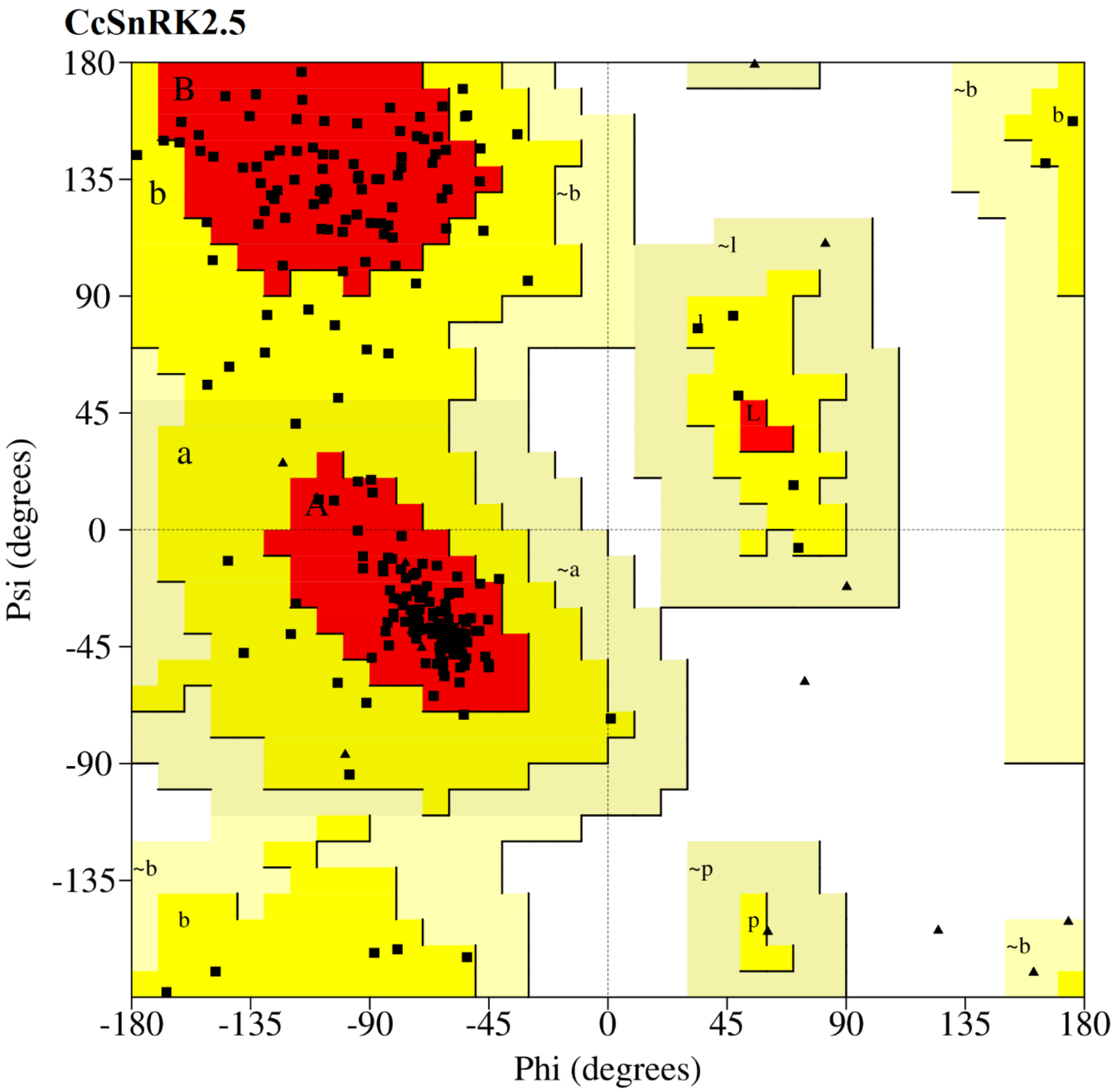


Plot statistics

| | | |
|---|---|---|
| Residues in most favoured regions [A,B,L] | 175 | 81.0% |
| Residues in additional allowed regions [a,b,l,p] | 41 | 19.0% |
| Residues in generously allowed regions [~a,~b,~l,~p] | 0 | 0.0% |
| Residues in disallowed regions | 0 | 0.0% |
| | ---- | ------ |
| Number of non-glycine and non-proline residues | 216 | 100.0% |
| Number of end-residues (excl. Gly and Pro) | 3 | |
| Number of glycine residues (shown as triangles) | 14 | |
| Number of proline residues | 16 | |
| | ---- | |
| Total number of residues | 249 | |

Based on an analysis of 118 structures of resolution of at least 2.0 Angstroms and R-factor no greater than 20%, a good quality model would be expected to have over 90% in the most favoured regions.

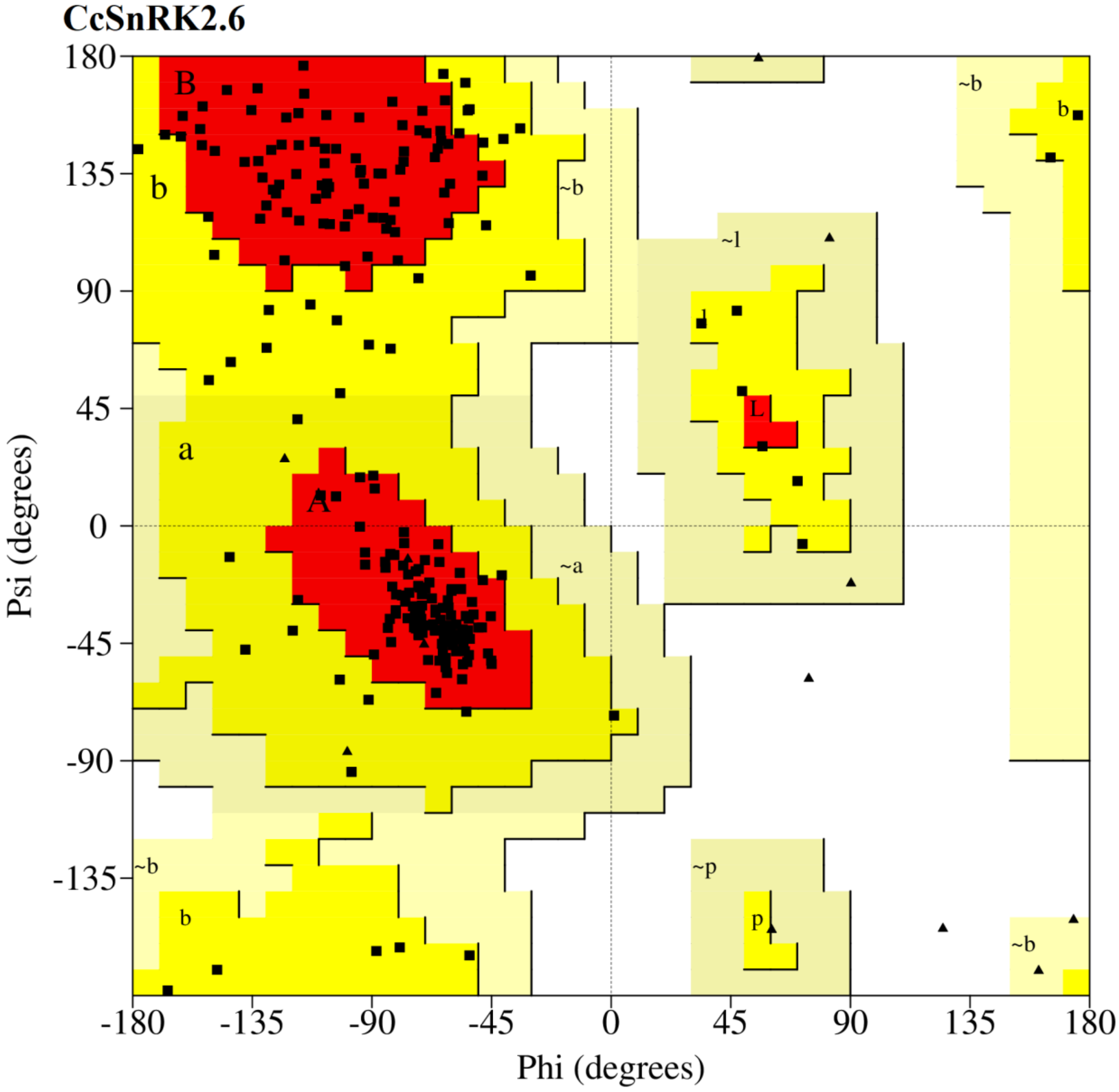


Plot statistics

| | | |
|---|---|---|
| Residues in most favoured regions [A,B,L] | 186 | 81.2% |
| Residues in additional allowed regions [a,b,l,p] | 43 | 18.8% |
| Residues in generously allowed regions [~a,~b,~l,~p] | 0 | 0.0% |
| Residues in disallowed regions | 0 | 0.0% |
| | ---- | ------ |
| Number of non-glycine and non-proline residues | 229 | 100.0% |
| Number of end-residues (excl. Gly and Pro) | 6 | |
| Number of glycine residues (shown as triangles) | 13 | |
| Number of proline residues | 15 | |
| | ---- | |
| Total number of residues | 263 | |

Based on an analysis of 118 structures of resolution of at least 2.0 Angstroms and R-factor no greater than 20%, a good quality model would be expected to have over 90% in the most favoured regions.

**CcSnRK2.7**

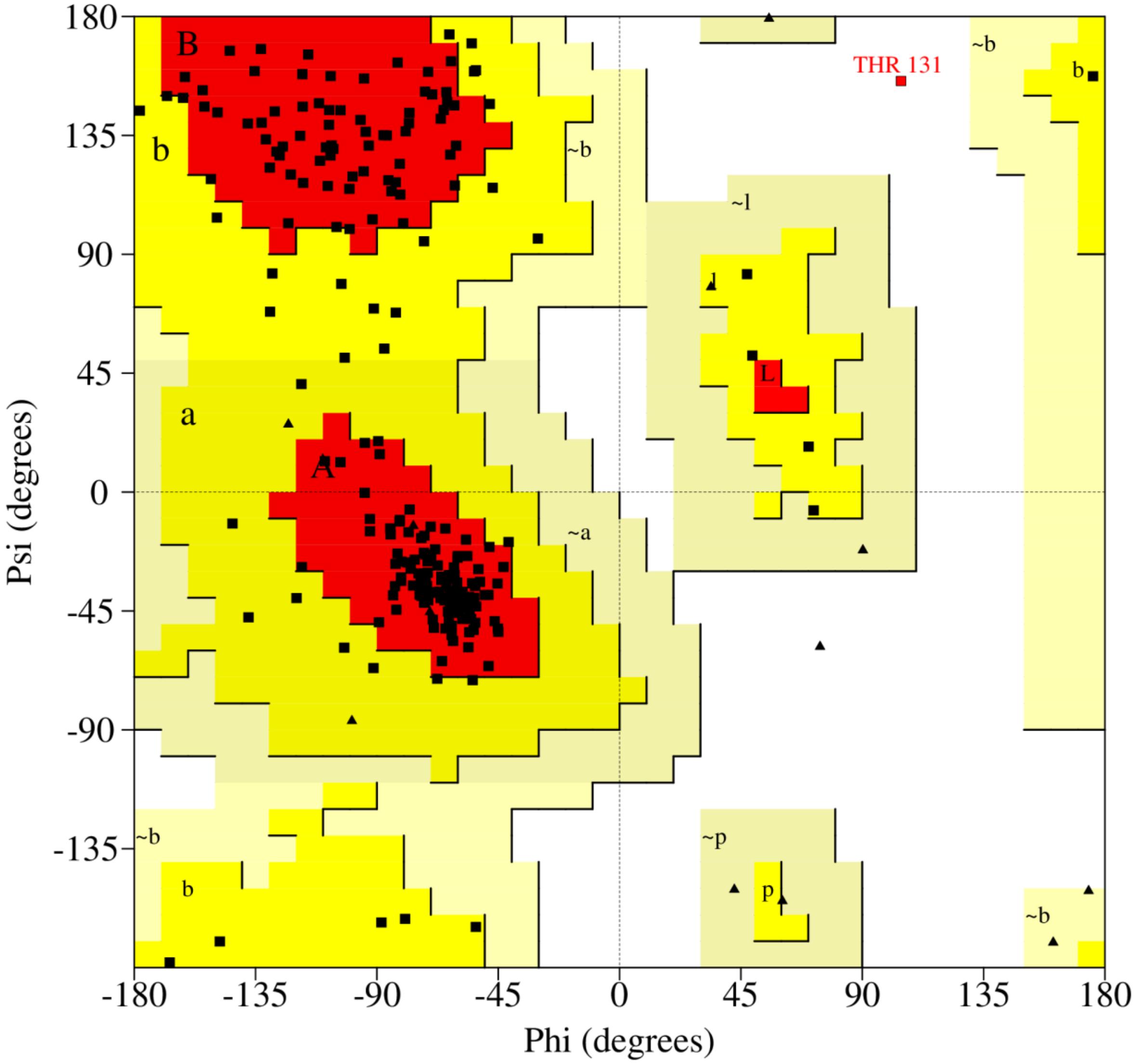


Plot statistics

| | | |
|---|---|---|
| Residues in most favoured regions [A,B,L] | 181 | 83.0% |
| Residues in additional allowed regions [a,b,l,p] | 36 | 16.5% |
| Residues in generously allowed regions [~a,~b,~l,~p] | 0 | 0.0% |
| Residues in disallowed regions | 1 | 0.5% |
| | ---- | ------ |
| Number of non-glycine and non-proline residues | 218 | 100.0% |
| Number of end-residues (excl. Gly and Pro) | 4 | |
| Number of glycine residues (shown as triangles) | 14 | |
| Number of proline residues | 14 | |
| | ---- | |
| Total number of residues | 250 | |

Based on an analysis of 118 structures of resolution of at least 2.0 Angstroms and R-factor no greater than 20%, a good quality model would be expected to have over 90% in the most favoured regions.

**CcSnRK2.8**

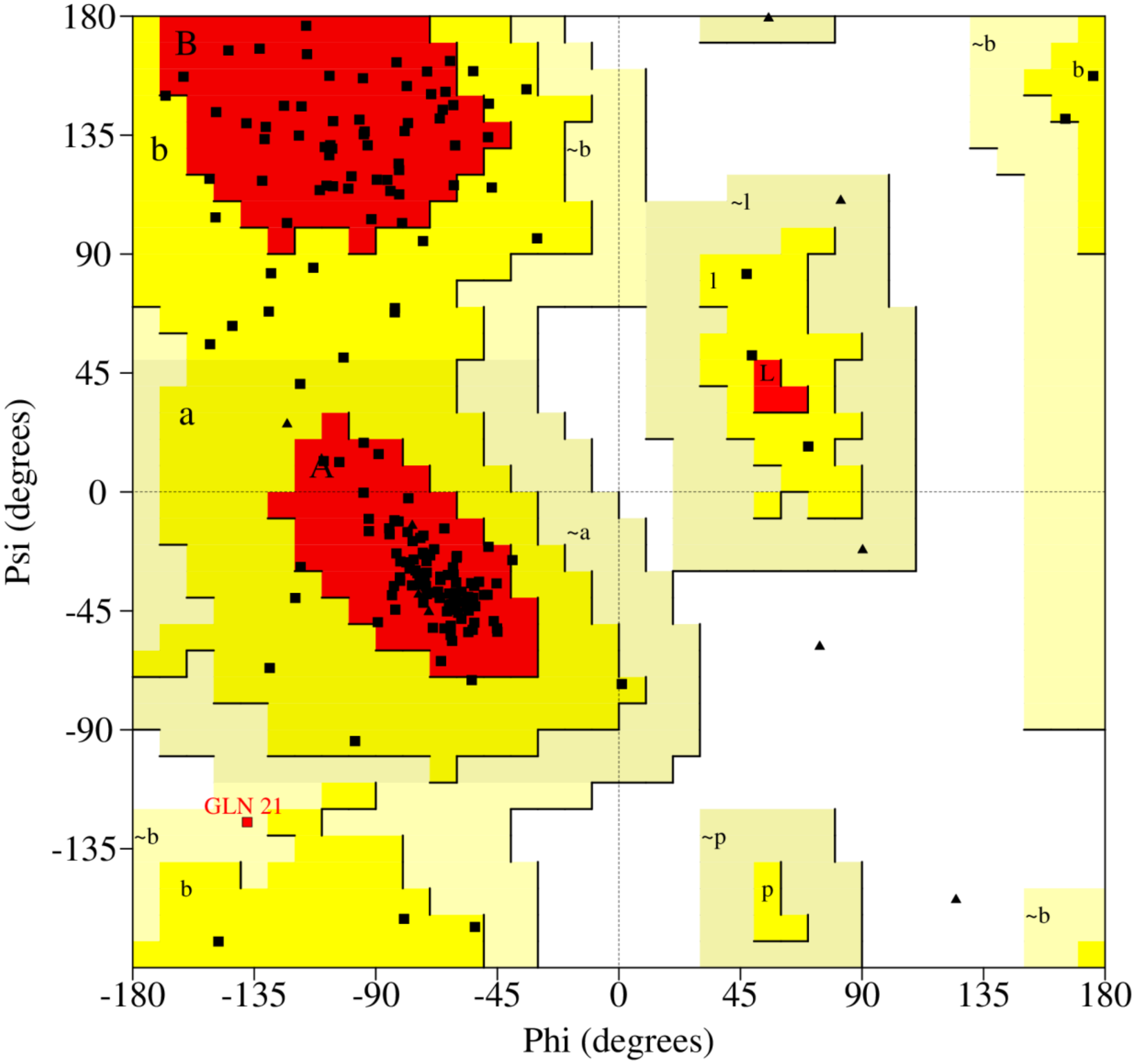


Plot statistics

| | | |
|---|---|---|
| Residues in most favoured regions [A,B,L] | 144 | 82.3% |
| Residues in additional allowed regions [a,b,l,p] | 30 | 17.1% |
| Residues in generously allowed regions [~a,~b,~l,~p] | 1 | 0.6% |
| Residues in disallowed regions | 0 | 0.0% |
| | ---- | ------ |
| Number of non-glycine and non-proline residues | 175 | 100.0% |
| Number of end-residues (excl. Gly and Pro) | 3 | |
| Number of glycine residues (shown as triangles) | 10 | |
| Number of proline residues | 12 | |
| | ---- | |
| Total number of residues | 200 | |

Based on an analysis of 118 structures of resolution of at least 2.0 Angstroms and R-factor no greater than 20%, a good quality model would be expected to have over 90% in the most favoured regions.
